# Bridging the Gap: Using Machine Learning Force Fields to Simulate Gold Break Junctions at Pulling Speeds Closer to Experiments

**DOI:** 10.1021/acsnano.5c11887

**Published:** 2025-11-13

**Authors:** William Bro-Jørgensen, Joseph M. Hamill, Davide Donadio, Gemma C. Solomon

**Affiliations:** † Department of Chemistry and Nano-Science Center, University of Copenhagen, Copenhagen Ø DK-2100, Denmark; ‡ Department of Chemistry, 8789 University of California Davis, Davis, California 95616, United States; § NNF Quantum Computing Programme, Niels Bohr Institute, University of Copenhagen, Copenhagen Ø DK-2100, Denmark

**Keywords:** machine learning force fields, single-molecule junctions, molecular electronics, gold nanowires, thermopower, Seebeck coefficient, break junctions

## Abstract

The properties and dynamics of gold nanowires have been studied for decades as an important testbed for several physical phenomena. Gold nanowires forming at contacts are an integral part of molecular junctions used to study the electronic and thermal properties of single molecules. However, the huge discrepancy in time scales between experiments and simulations, compounded by the limited accuracy of classical force fields, has posed a challenge in accurately simulating realistic junctions. Here, we show that machine-learning force fields uncover phenomena not captured by classical force fields when modeling Au–Au pulling junctions. Our simulations show a dependency of the average breaking distance on the pulling speed, highlighting a more complex behavior than previously thought. Our results demonstrate that the use of more accurate force fields to simulate metallic nanowires is essential for capturing the complexity of their structural evolution in break junction experiments. Our developments advance the modeling accuracy of molecular junctions, bridging the gap between experimental and simulation time scales.

## Graphical abstract

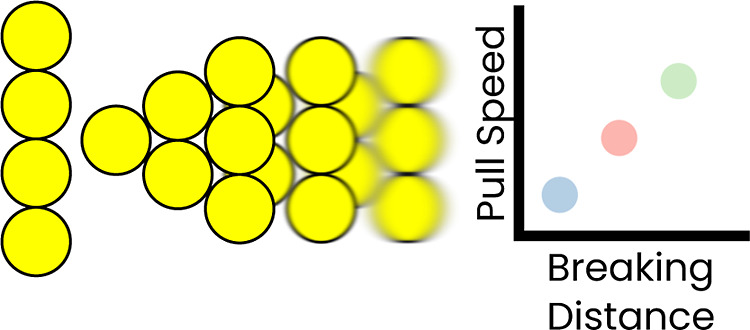

Throughout history, gold has been a symbol of value and a cornerstone in technological advancements; today, its manipulation at the atomic level, specifically through Au–Au junctions obtained by pulling metallic wires, enables the study of single-atom and single-molecule properties. Although seemingly simple, monometallic junctions have been studied for nearly three decades, offering a wealth of insights into the fundamental aspects of nanoscale physics.^1–4^ These junctions are not only central to fundamental studies of nanoscale physics, but also underpin a broad range of applications, including molecular electronics, single-molecule sensing, studies of plasmon phenomena in junctions, and atomic-scale device design.^5–12^ Given the growing interest in these systems across disciplines, a modeling framework capable of accurately simulating these experimental setups at experimentally relevant pulling speeds is timely and of broad relevance. Yet, a knowledge gap still persists regarding the dynamics of Au–Au junctions as molecular simulations of these systems out of equilibrium are challenged by the need to tackle very long time scales with highly accurate models.

Early atomistic simulations established the now accepted picture of necking, atomic-chain formation, and stepwise conductance in elongating Au nanocontacts, albeit at strain rates and with force fields constrained by their computational limits.^13–15^ Specifically, pulling junctions in silico at the same (slow) speed as experiments remains a formidable task and most computational studies use pulling speeds from 10^9^ nm/s to 10^5^ nm/s.^16–24^ In contrast, typical scanning tunneling microscope break junction (STM-BJ) and mechanically controllable break junction (MCBJ) experiments use pulling speeds between 10 and 50 nm/s. To pull a junction for 20 Å at 20 nm/s would require 0.1 s of simulation. As each time step needs to be approximately 10^–15^ s (1 fs), such long simulations are currently outside the limit of what can be simulated in a reasonable wall clock time-even with classical force fields and state-of-the-art GPUs.

This discrepancy raises questions regarding the comparability of in silico results to experimental findings. It has previously been argued that if the pulling speeds in molecular dynamics (MD) calculations are kept slow enough to get reversible behavior, the sampled structures throughout the trajectory should be independent of pulling speed, and as a result, any derived properties from these structures should also be independent of pulling speed.^25,26^ While it may be true for certain stages of the pulling experiment, this hypothesis overlooks the inherent nonequilibrium nature of the junction at the point of rupture along with any slow processes that happen on a time scale longer than the time scale of the pulling, as originally pointed out by Todorov and Sutton.^16^

Several experimental studies have examined the influence of pulling speed and temperature on the formation and breaking of gold nanowires by measuring the electronic quantum of conductance (1 G_0_). Pobelov et al.^27^ performed atomic force microscope (AFM) pulling experiments at room temperature, finding that Au nanocontacts typically break spontaneously at force loading rates of 2.6 × 10^–6^ N/s or lower. Their observations suggest that room-temperature measurements of 1 G_0_ correspond to point contacts rather than the linear chain-like structures observed at cryogenic temperatures. This conclusion is supported by Kamenetska et al.,^28^ who found that Au nanocontacts melt into a smoother geometry at room temperature compared to the more corrugated structures observed at cryogenic temperatures.

Huang et al.^29^ explored a range of pulling speeds from 0.4 to 344 nm/s in STM-BJ experiments. They found that the length of the 1 G_0_ plateau increases slightly from ∼10 Å to ∼17 Å when increasing the pulling speed from 7 to 54 nm/s. However, at higher or lower pulling speeds, the length of the plateaus remained independent of pulling speed. They also found that the conductance of the 1 G_0_ plateau is independent of the pulling speed across the entire tested range. In contrast, Tsutsui et al.^30^ found that at a pulling speed of 8 nm/s and higher the length of the conductance plateau is independent of pulling speed and that from 0.8 nm/s to 8 × 10^–4^ nm/s the length of the conductance plateaus continues to shorten. Similar results were found by Dyer and Monti.^31^ Alwan et al.^32^ have looked at speeds from 1 nm/s all the way up to 1000 nm/s focusing on the lifetimes of 1 G_0_ plateaus rather than the breaking distance. Their findings align with the above-mentioned studies, showing a reduction in 1 G_0_ plateau lifetimes with increased pulling speeds. These experiments (summarized in Table 1) indicate that while the conductance at the 1 G_0_ plateau may not be directly affected by the pulling speed, the geometry of the rest of the junction is likely influenced by it. This includes the shape and structure of the gold electrodes beyond the single-atom contact. In molecular break junction experiments, such geometric differences—such as whether the resulting gold pyramid is sharp and elongated or short and wide—can affect both how likely and in which way a molecule will bridge the electrodes. Therefore, models aiming to simulate these experiments should accurately capture the formation and breaking dynamics of Au nanocontacts, including their full geometric evolution.

**Table 1:** Effect of Pulling Speed on Au Nanocontacts

study	method	T	speed (nm/s)	key findings
Pobelov et al.^27^	AFM	RT	-	slow loading can lead to spontaneous rupture; at low temperatures, G_0_ traces likely reflect long atomic chains
Kamenetska et al.^28^	STM-BJ	cryo → RT	15	RT geometries are smoother; cryogenic conditions yield more corrugated/sharp structures
Huang et al.^29^	STM-BJ	RT	0.4–344	1 G_0_ plateau length increases modestly from ∼10 Å to ∼17 Å over 7 → 54 nm/s; the length of the plateaus remains independent of the pulling speed at higher or lower pulling speeds
Tsutsui et al.^30^	STM-BJ/MCBJ	RT	8 × 10^–4^ to 8	for ≥8 nm/s, 1 G_0_ plateau length is approximately speed-independent; from 0.8 down to 8 × 10^–4^ nm/s, plateaus continue to shorten
Dyer and Monti^31^	MCBJ	RT	10^–3^ to 10	reports a similar trend as Tsutsui et al.: plateau length shortens at very low speeds and is broadly speed-independent at higher speeds
Alwan et al.^32^	STM-BJ/MCBJ	RT	1–1000	1 G_0_ lifetimes decrease with increasing pulling speed (faster stretching → shorter survival at 1 G_0_)

In this study, we show how a machine learning potential (MLP) trained on first-principles data can be leveraged to perform MD simulations of Au break junctions at pulling speeds closer to those observed experimentally at a quasi-first-principles accuracy. We assess the ability of the chosen MLP, the neuroevolution potential (NEP) model,^33^ to reproduce density functional theory (DFT) forces during nonequilibrium MD simulations of Au break junctions. We show that MD simulations with NEP provide very different structural and dynamical features of Au break junctions compared to empirical models, such as the Embedded Atom Model (EAM)^34^ and ReaxFF,^35^ commonly used for these kind of studies. NEP simulations are in substantially better agreement with STM-BJ experiments, as they capture the dependence of the breaking behavior as a function of the pulling speed. Moreover, a comparison of structures from the NEP model simulations with experimental data shows qualitative agreement. Notably, our simulations show the formation of pyramidal structures in the amorphous region just prior to breaking, as well as the emergence of helical structures along the nanowire.

## Results and Discussion

We start by evaluating the performance of the trained NEP model on holdout data (configurations withheld from both training and validation) of gold break junctions at the size scale used in our nonequilibrium simulations. In particular, we want to evaluate the accuracy of the NEP in reproducing the forces on the gold wire formed as the electrodes are pulled apart. For this evaluation, we ran two new trajectories with a pulling speed of 10^–7^ Å/fs using the trained NEP model. Other settings remained the same as for production runs. These trajectories featured larger electrodes (8 × 8) than those used during training (6 × 6) and there were no 4,4′-bipyridines present. We evenly sampled 117 new structures from these two trajectories and calculated the forces on each of these structures using DFT, EAM, ReaxFF, and NEP.

The plots in Figure 1A compare the force components in all directions (*x*, *y*, and *z*) on all atoms for all structures. In the legends of each subplot, we list the mean absolute error (MAD) and the root mean squared error (rmsd) for each force field versus DFT reference data. The first column plots the forces from DFT versus the forces from the potential. In the second column, we plot the predicted force from the given potential versus the error of that potential. The error is calculated as the simple difference between two components. Figure 1A shows that the NEP model generally reproduces the DFT forces much more accurately than both EAM and ReaxFF. Not only is the MAD substantially smaller, but NEP forces correlate with DFT over the whole range of values. Respectively, ReaxFF overestimates and EAM underestimates the values of the force components in the tails of the distribution. Though the NEP model reliably reproduces the majority of the forces, we observe a set of force values with systematic errors that lay outside the main distribution. These forces correspond to the surface gold atoms which are less accurately treated by the NEP model. However, these atoms are not critical to accurately simulate the pulling experiment the formation of wires, and the breaking of the junction.

**Figure 1:**
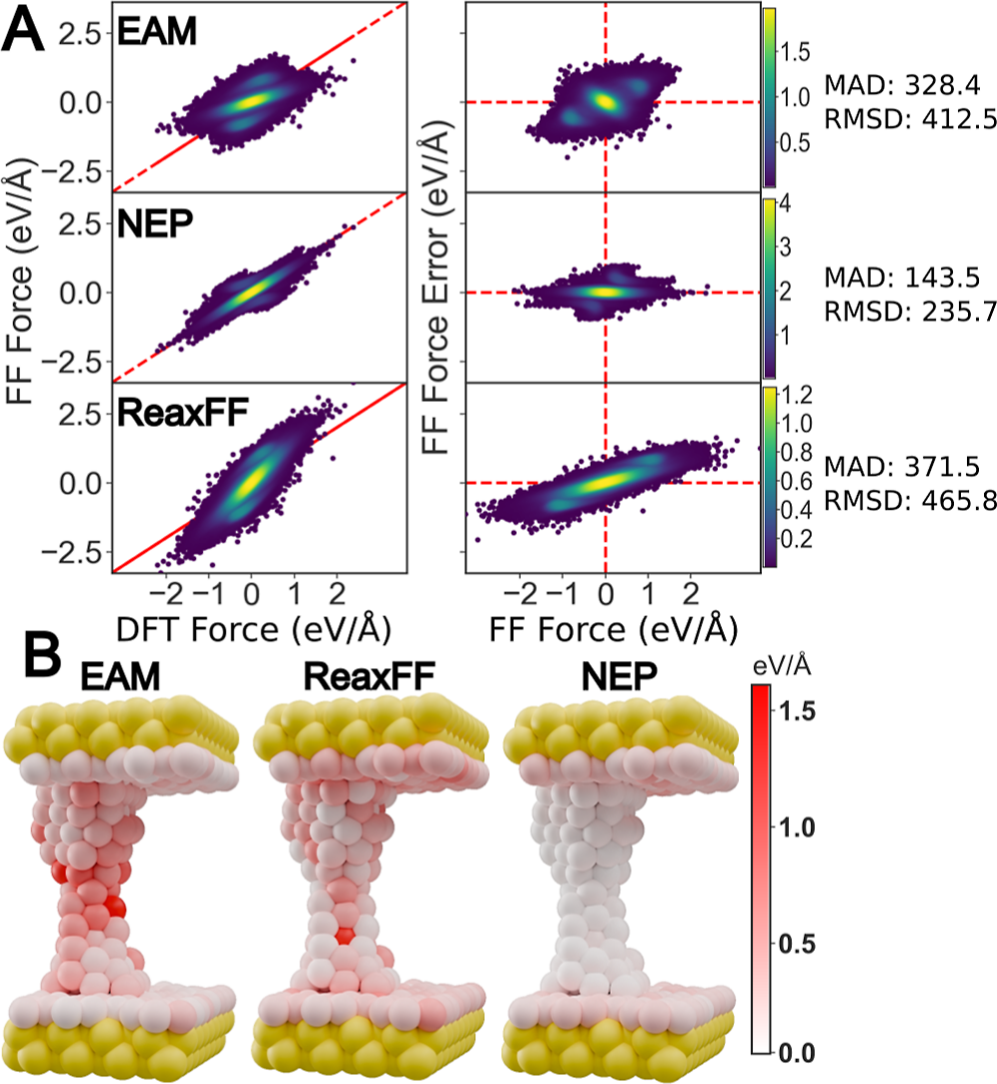
Error analysis of forces on the holdout structures. (A) Plot of the forces from the three models versus the forces from DFT for each *x*-, *y*-, and *z*-component. Gaussian kernel density estimation has been used to color overlapping points—lighter color corresponds to higher density. MAD and rmsd for each model is shown to the right of each subplot. The units are meV/Å. (B) Visualization of error magnitudes on each atom in the central region for EAM, ReaxFF, and NEP for a single holdout structure. The yellow spheres are frozen Au.

Figure 1B, provides an atom-by-atom color-coded representation of the absolute error on the forces from a single structure, defined as $\epsilon =\sqrt{\sum_{i}{\left(f_{i}^{FF}-f_{i}^{DFT}\right)}^{2}}$ , where *f*_*i*_^FF^ and *f*_*i*_^DFT^ are the *i* force components computed by the three potentials and by DFT, respectively. A single frame is shown but it is representative of the general trends: our NEP model is much more accurate than the other empirical potentials for the atoms in the bridge between the two electrodes. Conversely, all models exhibit similar errors on the gold atoms at the electrode surface. These features can be quantified by computing the error metrics on the bridge atoms alone. If we exclude the contribution from the flat surface of the electrodes MAD and rmsd drop substantially to 78.8 and 96.0 meV/Å for NEP, but increase for EAM (403.3 and 490.3 meV/Å) and drop slightly for ReaxFF (317.5 and 400.2 meV/Å). We note that EAM is particularly accurate at modeling the Au surface. In contrast, our NEP model is trained for effective performance on pulling junctions which includes both disordered and crystalline regions.

However, since energies and forces are often insufficient to assess the quality of MLPs,^36^ it is desirable to benchmark the NEP model against physical observables. As the system of interest includes gold electrodes, we need an accurate description of the dynamics of bulk gold. To verify the capability of the NEP model in this regard, we computed the phonon dispersion of fcc gold using NEP and the two classical force fields, comparing against DFT and coherent inelastic neutron scattering data.^37^ All calculations were performed on an 8 × 8 × 8 replica of Au fcc primitive cell employing the frozen phonon approach, with atomic displacements of 0.01 Å. A DZP basis set and the PBE functional were utilized for the DFT calculations, ensuring convergence of energy changes to below 10^–9^ eV/electron and density changes to below 10^–9^ electrons/(valence electron). Except for a slightly tighter energy and density convergence, these are the same settings used when generating the training data. We used a lattice constant of 4.169 Å.

Figure 2 shows the phonon dispersion for bulk Au as calculated by the three different potentials alongside DFT for comparison: DFT (black dashed line); the ReaxFF potential (blue line); the EAM potential (orange line); and the NEP model (green line). These results are compared with room-temperature phonon dispersion of gold measured experimentally. The phonon dispersions from both DFT and NEP align closely with the experimental results. This agreement shows that the NEP model excellently reproduces the bulk part of the gold electrodes and that the chosen level of DFT is appropriate for an accurate description of bulk Au. The EAM potential also reproduces well the phonon dispersions of bulk gold. Conversely, ReaxFF overestimates the stiffness of gold metallic bonds resulting in too high frequencies for both acoustic and optical phonon branches.

**Figure 2:**
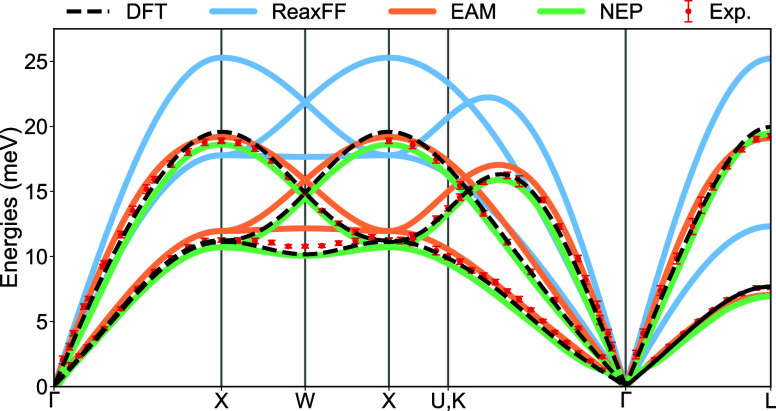
Phonon dispersion comparison for Au across different methods. DFT results are depicted by a black dashed line, the EAM results by an orange line, the NEP results by a green line, and the blue line is the results from ReaxFF. Experimental phonon dispersion at room temperature are represented by red dots, with uncertainty indicated by horizontal bars.^37^

After ascertaining the performance of NEP to reproduce the structural and vibrational properties of bulk gold and the DFT forces of bridging gold electrodes, we now perform a series of nonequilibrium MD simulations of Au–Au break junctions. We start by focusing on the breaking distances of the Au–Au junctions under varying pulling speeds to understand the junction evolution dynamics with different models. We plot 1D-histograms of the distance of rupture at each pulling speed in Figure 3. The 1D-histograms are categorized by the pulling speed and the force field used: NEP model (top plot), EAM potential (middle plot), and ReaxFF potential (bottom plot). The error bars on the counts in the 1D-histogram are assumed to follow a Poisson distribution and are calculated as $\sqrt{n}$ where *n* is the count in each bin. For each pulling distribution, we fit a Poisson distribution function to quantify their mean breaking distance (dashed lines in Figure 3). The histograms in Figure 3 are normalized by dividing each histogram by its tallest bin.

**Figure 3:**
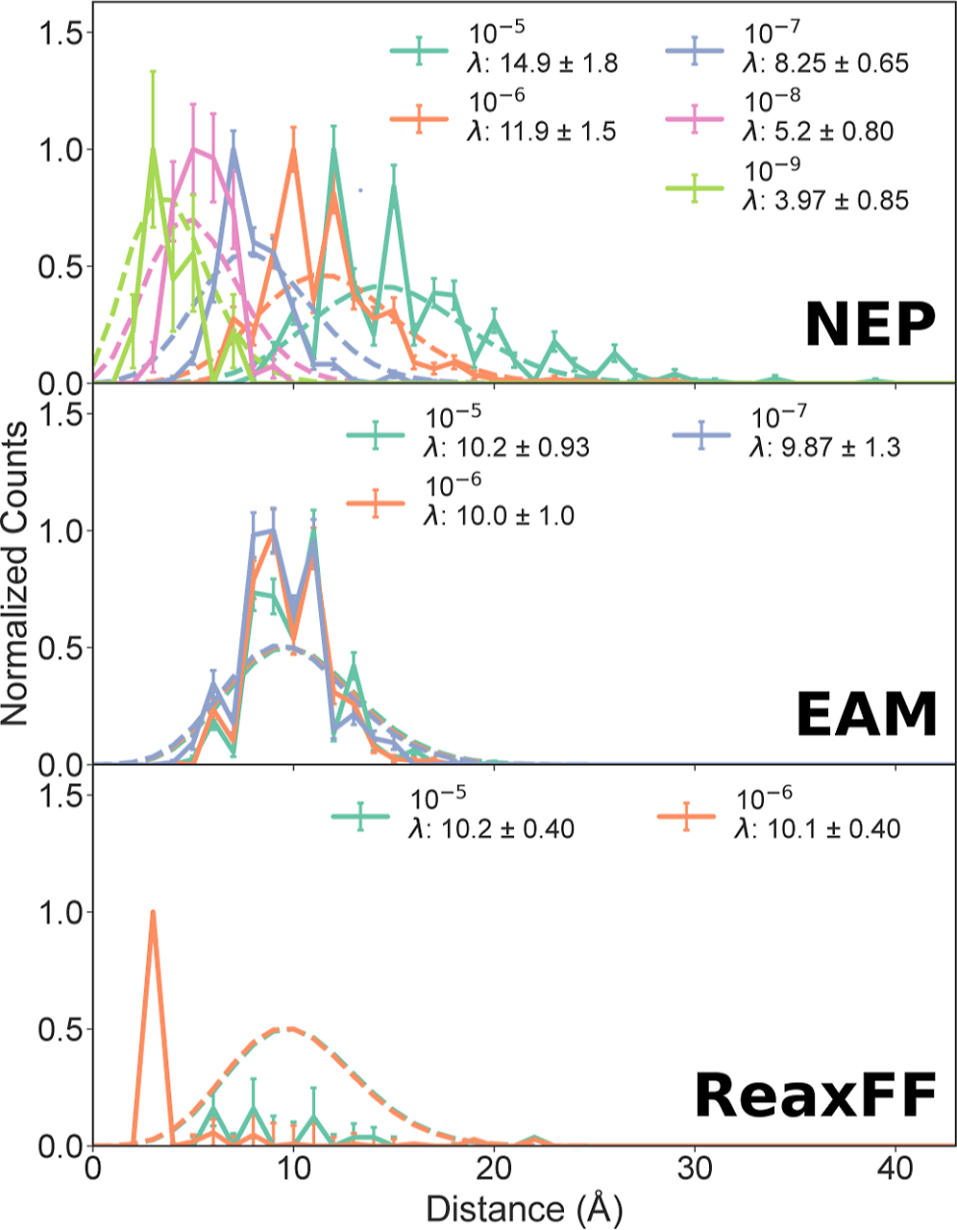
Breaking distance comparison for Au–Au junctions simulated at different pulling speeds at 300 K. The pulling speeds are 10^–5^ Å/fs (green), 10^–6^ Å/fs (orange), 10^–7^ Å/fs (gray), 10^–8^ Å/fs (pink), and 10^–9^ Å/fs (light green). The top, middle, and bottom plots correspond to simulations using the NEP model, the EAM potential, and the ReaxFF potential, respectively. Dashed lines represent Poisson fits with parameters λ (mean) indicated in the legend. The uncertainty of the counts in each bin is assumed to follow a Poisson distribution. Bin width 1 Å.

We first notice that ruptures happen at multiples of approximately 2.6 Å, slightly shorter than the Au–Au equilibrium distance in the bulk, for all potentials, especially for NEP and EAM (bin width 1 Å). This pattern of rupture at consistent intervals has been observed experimentally,^3,38,39^ and the 2.6 Å step is in agreement with recent measurements.^40^ For the NEP model, especially at 10^–9^ Å/fs (light green line), the distinct peaks blur, possibly due to the limited sample size (22 samples) preventing clear resolution of the individual peaks.

Interestingly, we observe a pronounced dependence of the breaking distance on pulling speed with the NEP model, suggesting that even at the lowest pulling speed the system is not in quasi-equilibrium, contrary to what might be inferred from simulations using the EAM and ReaxFF potentials. This dependence is similar to what was seen in MD simulations using an effective medium potential.^27^

In Section 13 of the Supporting Information, we show results where the atoms in the electrodes are pulled by a spring force constant with a force constant of *k* = 0.6242 eV/Å^2^, which matches experimental force constants.^27^ We use a time-step 0.0075 ps and only apply the thermostat to the atoms in the 2D periodic part of the electrodes. We plot the breaking distance distributions in Figure S13. We replicate the same pulling speed dependence as seen in Figure 3.

The observation that the Au–Au junction breaking distance depends on pulling speed when using the NEP model diverges from the expected behavior where results should ostensibly be independent of pulling speed, as reported in previous studies using different simulation potentials.^25,26^ Consequently, the results from the NEP model indicate that if it were possible to use DFT for the full trajectory—which the NEP model is a proxy for—we should observe nonequilibrium behavior.

From these observations of the dependence of the breaking distance on pulling speed, we now wonder: how do these dynamics manifest in the physical structure of the Au–Au junctions? To answer this question, we qualitatively assess the Au–Au junctions from NEP-model MD trajectories just before rupture. Figure 4 shows the shortest and longest breaking structure at three different pulling speeds: 10^–5^, 10^–7^, and 10^–9^ Å/fs. As expected, intuitively and from the previous section, rapidly pulling the Au–Au junctions results in long, single-atom gold nanowires being pulled out while slower pulling speeds reduce the maximum pulling distance. Interestingly, structures from the shortest pulls are qualitatively similar, with junctions consisting of one to three Au atoms. This consistently lower minimum breaking distance suggests a threshold thinning of the junctions is necessary before rupture. Additionally, all observed structures retain the pyramidal shape of the Au electrode, consistent with high-resolution transmission electron microscopy (HRTEM) images of electron-beam-grown gold nanowires.^41^

**Figure 4:**
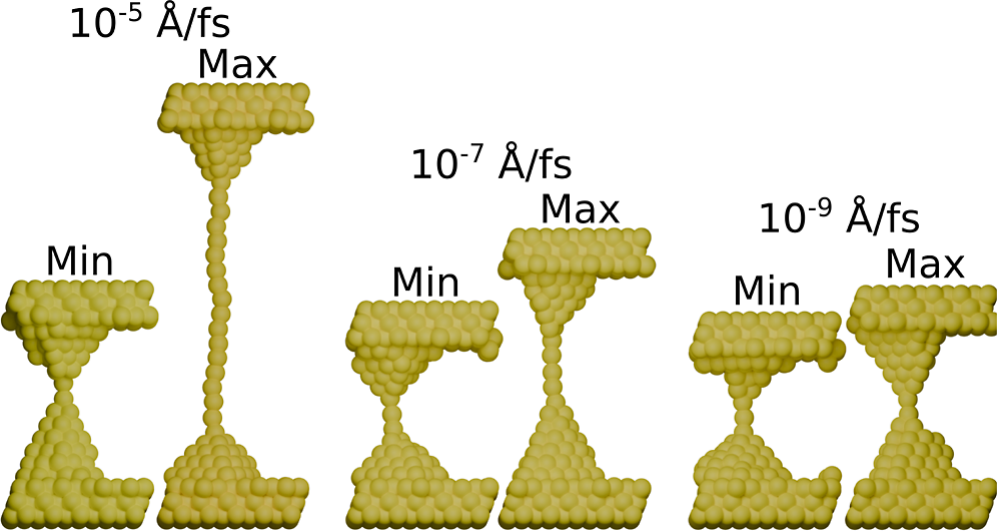
Qualitative comparison of Au–Au junctions under varying pulling speeds. We illustrate three pairs of Au–Au structures, each subjected to different pulling speeds. The left pair is pulled at a speed of 10^–5^ Å/fs, the middle pair at 10^–7^ Å/fs, and the right pair at 10^–9^ Å/fs. The depicted stages represent the moment just before junction breakage, capturing both the minimum (left) and maximum (right) distances pulled.

These qualitative differences will impact any subsequent analysis of these Au–Au junctions, particularly those break junctions where molecules are incorporated. Only if the description of the pure Au–Au junction is modeled qualitatively correctly, we can hope to capture the experimental variance seen in single-molecule break junctions.

Another notable similarity between the pulling junctions from the NEP model and experiments is the appearance of helical structures in the amorphous region. Similar helical structures have been observed in experimental studies of Au nanowires.^42^ In Figure 5, we show two selected structures from simulations using the EAM potential (left side of Figure 5) and the NEP model (right side of Figure 5). The Au atoms comprising the helical structure are colored in shades of blue to highlight the helix. These snapshots are from simulations conducted at the slowest pulling speeds: 10^–7^ Å/fs for the EAM potential and 10^–9^ Å/fs for the NEP model. As the simulations with the NEP model are pulled a shorter distance before breaking than for the EAM potential, the helix is correspondingly shorter. Note that these helical structures were not observed in simulations using the ReaxFF potential; therefore, they are excluded from the figure. Larger, multishell helices have previously been investigated with the EAM potential.^43^ The ability of the NEP model to replicate these helices further demonstrates its effectiveness reflecting expected physical phenomena.

**Figure 5:**
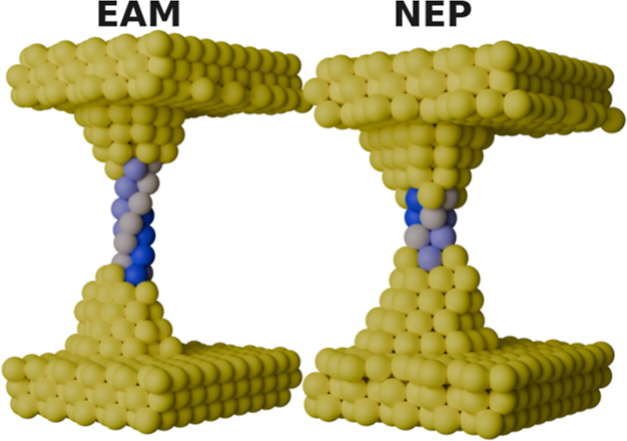
Illustration of gold forming a helix when using the EAM force field with a pull speed of 10^–7^ Å/fs (left) and the NEP model with a pull speed of 10^–9^ Å/fs (right). All atoms are Au, but some have been colored in shades of blue to highlight the helical structure.

In Section 14 of the Supporting Information, we include illustrations of individual pulling force traces from the simulations with a spring force attached to the bottom electrodes. Similar to what is seen in experiments, the force–distance curves has a steeper slope in the beginning of the pull and flattens as rupture approaches.^16,19^

In Section 15 of the Supporting Information, we analyze rupture forces as a function of loading rate following the protocol of Pobelov et al.^27^ Their study was motivated by the puzzling observation that gold nanowire breaking forces remained essentially unchanged across experiments conducted under different conditions. Using MD simulations, they showed that, under slow loading, thermal fluctuations can trigger spontaneous bond scission, whereas rapid loading suppresses these fluctuations and thus leads to a breaking force vs loading rate correlation. In our analysis, due to our larger system size, we cannot simulate a low enough loading rate to reach a regime where spontaneous rupture occurs. This observation provides further evidence that even the slowest pulling speeds are not converged.

Direct comparison of breaking distances with experimental data is challenging due to differences in measurement techniques. Experimental measurements quantify the breaking distance by estimating the length of the 1 G_0_ plateaus, while our simulations directly assess the distance to rupture interatomic bonds. For a more direct comparison, we need to examine the 1 G_0_ plateau lengths from transmission calculations, acknowledging that experimental setups might still allow for longer pulling distances due to a larger amount of Au being present in the central region. Hence, we calculate the conductance within the NEGF framework. Whereas there are well-known shortcomings in using DFT to calculate transmission in molecular junctions, the standard approximations of DFT are justified for systems such as chains of Au atoms.^44^ As the calculations are computationally expensive, we only calculate transmission for a randomly selected subset of 10 MD trajectories for each pulling speed. Note that these trajectories are not necessarily the same trajectories that had the longest/shortest pulling distance as was shown in Figure 4.

Figure 6 shows the transmission of Au–Au junctions at different pulling speeds for all three potentials. For each potential and pulling speed, we display three selected traces. The rows correspond to the NEP model (top plot), the EAM potential (middle plot), and the ReaxFF potential (bottom plot), while the columns from left to right show the transmission at decreasing pulling speed. The final column on the far right display a 1D histogram of all calculated traces for each potential. As transmission calculations are computationally expensive, we omit the initial portion of the traces. The *x*-axis in Figure 6 represents the distance pulled from the first image to the final image where the transmission reaches zero. Each trace is offset horizontally for visual clarity.

**Figure 6:**
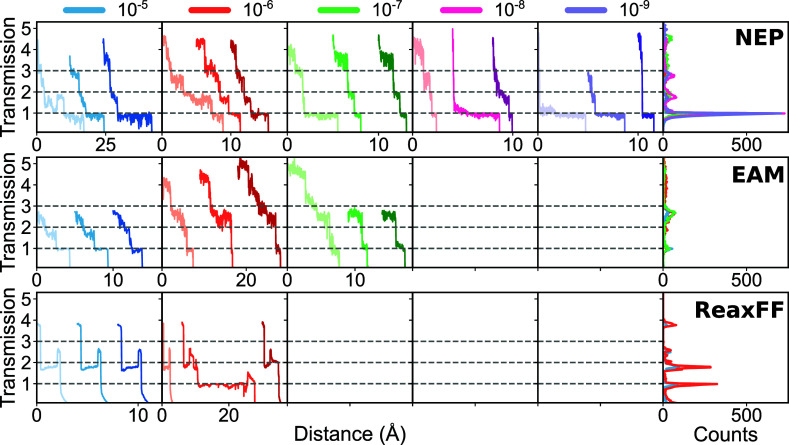
Electronic transmission of Au–Au junctions at various pulling speeds for three different potentials. The rows correspond to the NEP model (top plot), the EAM potential (middle plot), and the ReaxFF potential (bottom plot). Each column, from left to right, shows the transmission for three selected traces, with the final column displaying a 1D histogram of all traces in each data set. Horizontal gray dashed lines indicate integer multiples of G_0_. Note the different *X*-axes. Plots without traces indicate speeds at which pulling simulations were not performed.

Notably, the transmission traces from the NEP model and the EAM potential exhibit noise which seems to be mostly capped at integer values of G_0_. This upper limit is consistent with the fact that one-atom chains of Au only have one open transmission eigenchannel, dominated by its s valence orbitals.^45–47^ Furthermore, because there is no experimental noise or averaging involved, it is less likely for the transmission noise to exceed these integer values of G_0_. In contrast, the traces from the ReaxFF potential display less noise due to their more uniform breaking.

As mentioned earlier, we can only compare the lengths of the 1 G_0_ plateaus if we wish to compare with the breaking distances reported from experiments. The length of the plateaus for the traces from the NEP model pulled at 10^–9^ Å/fs and at 300 K are between 0.5632 Å and 4.576 Å with a mean of 1.832 Å. This is shorter than the average lengths reported for break junction experiments conducted at 4.2 K although a substantial fraction of the experimental traces break at 2.5 Å or shorter,^3,38–40^ and matches what is seen in experiments performed at room temperature.^28,29^ For the EAM potential at a pulling speed of 10^–7^ Å/fs and 300 K, the 1 G_0_ plateaus are between 0.0780 Å and 1.43 Å with a mean of 0.738 Å. This average plateau length is significantly shorter compared with experiments.

In the 1D-histogram, both the NEP model and the EAM potential exhibit peaks at 1 G_0_ and less clear plateaus at 2 and 3 G_0_. This observation matches experimental findings.^32,40,48–50^ But, unlike the traces from the EAM potential, the NEP model replicates some subtle experimental characteristics. For example, the second trace at 10^–8^ and the first trace at 10^–9^ Å/fs exhibit features consistent with parity oscillations as has also been seen in experiments.^30,39,51^ In these traces, the parity oscillation appears as periodic fluctuations in the transmission signal, where brief segments exceed a transmission value of 1, followed by a slight drop below 1. In Section 10 of the Supporting Information, we illustrate the junction geometry in the middle of the parity oscillation and just after it goes back to 1 G_0_. The structure at slightly above 1 G_0_ is characterized by a single gold adatom bridging two pyramidal electrodes, whereas the 1 G_0_ plateau corresponds to a short, linear gold chain connecting the tips. Such gold structures with noninteger conductance states have been theoretically and experimentally explored previously.^52^

Transmission traces of ReaxFF predominantly lack the 1 G_0_ plateau, observed only for one trajectory at 10^–6^ Å/fs (see the middle red trace in the bottom plot). This discrepancy, along with atypically sharp increases in the transmission prior to rupture, suggests limitations of ReaxFF in accurately modeling the evolution of Au–Au junctions. Such atypical behavior, generally absent in experimental data, points to the need for careful reconsideration of the use of ReaxFF for these simulations.

In Section 12 of the Supporting Information, we include a representative push–pull trajectory for a junction bridged by 4,4′-bipyridine (44BPY), and compute the conductance during the push phase as the electrodes are brought almost back into contact.

As 44BPY transitions from a fully stretched to a tilted geometry, the transmission increases smoothly with compression, without the distinct switching between high- and low-conductance states often reported in experiments.^53^ This continuous transition implies that the commonly invoked mechanism for those discrete states may be more complex than originally thought. Notably, experimental studies that measure conductance during compression tend to report more pronounced conductance shifts.^54,55^ We will explore these nuances in detail in a forthcoming publication.

In Section 17 of the Supporting Information, we show results of push–pull simulations with pure gold junctions. We show distributions of the distance it takes to push junctions together after they have ruptured (Figure S18A) and the correlation between the breaking and reformation distances (Figure S18B).

On average, the reformation distances in our simulations are slightly longer than those observed experimentally.^28^ This may be due to the much faster pulling rate in our simulations, which likely limits tip relaxation and melting compared to experiments.

Finally, we calculate the thermopower of the gold pulling junctions as an additional physical observable measured in break junction experiments. Using eq 1, we compute the thermopower from the electronic transmission for each potential and each pulling speed. The thermopower is calculated using the same frames as those for the electronic transmission in Figure 6. The resulting distributions are shown in Figure 7.

**Figure 7:**
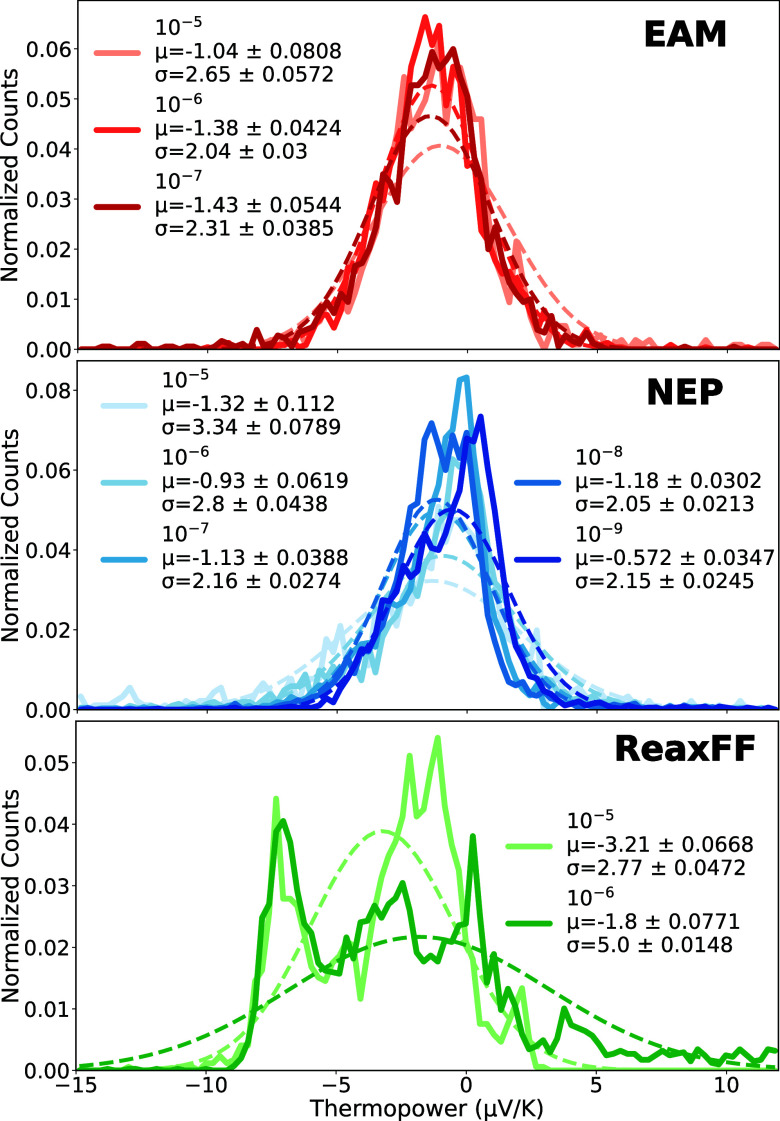
Distributions of thermopower for each potential at each pulling speed. The rows correspond to the EAM potential (top plot, red solid line), the NEP model (middle plot, blue solid line), and the ReaxFF potential (bottom plot, green solid line). Each pulling speed is represented by an increasingly darker shade. A Gaussian fit to each distribution is shown with a dashed line in the corresponding color. Fit parameters (mean and standard deviation) are included in the legend.

Both EAM and NEP predict average thermopower values of approximately −1 μV/K, consistent with experimental observations at room temperature^56,57^ and at cryogenic temperatures.^58^ We note that other experiments find the thermopower to fluctuate around 0, resulting in an average of 0 μV/k.^59–61^ An exception is observed for ReaxFF at 10^–5^ Å/fs, which yields an average thermopower of approximately −3 μV/K.

EAM yields a symmetric, nearly Gaussian thermopower distribution, while NEP shows a slight skew with a tail toward negative values. Both resemble experimental distributions.^57^ In contrast, ReaxFF produces a multimodal distribution that does not match experimental data.

In Section 11 of the Supporting Information, we present 2D-histograms of the conductance versus thermopower for each potential at each pulling speed. Although sample size is limited, the EAM potential and the NEP model both show a minimum in thermopower when the conductance is near integer values. This correlation is consistent with experimental results.^57^ The ReaxFF potential does not show the same correlation.

The NEP model yields results that align well with experimental observations that find the thermopower to be negative. As noted by Matsushita et al.,^60^ these differences can arise from the presence or absence of junction defects, meaning that our model systems are potentially constructed in closer alignment with the experiments reporting negative thermopower. While similar agreement is seen for the EAM potential, only the NEP model can be readily extended to include molecular components.

## Conclusions

In summary, we have explored the evolution of Au–Au pulling junctions with different potentials across a range of pulling speeds. Specifically, we compared the performance of an ML-based force field called NEP with two commonly used classical force fields, the EAM potential and the ReaxFF potential. First, we performed an extensive evaluation of the performance of the NEP model on Au–Au junctions comparing its results to both DFT and experimental data. The NEP model outperformed the classical force fields across all tested metrics, particularly excelling in accurately reproducing the experimental phonon dispersion relations of bulk Au. Next, we examined the relationship between breaking distance and pulling speed for the three potentials. Consistent with previous studies, we found that the classical force fields show minimal dependence on pulling speed, with this dependence becoming saturated at 10^–5^ Å/fs. In contrast, the breaking distance of traces from the NEP model displayed a strong dependence on pulling speed. Even at a slow pulling speed of 10^–9^ Å/fs, we did not reach an equilibrium regime, indicating a substantial sensitivity to pulling speed.

To validate the ability of our simulations to replicate important experimental features, we compared several structural and electronic features with experimental observations. A qualitative comparison of Au–Au structures from the NEP trajectories at various pulling speeds revealed that the possibility of forming very long single-atom chains at faster pulling speeds. However, when the pulling speed was reduced to 10^–9^ Å/fs, the longest structures observed consisted of only one or two pulled-out Au atoms. These chains are consistent with conductance measurements that associate 1 G_0_ plateaus with single-atom contacts. The pyramidal electrode geometry seen immediately before rupture matches HRTEM images of gold nanowires in junctions. Additionally, we find helical atomic structures in the amorphous region of the junction, a feature also reported in experimental studies of gold nanowires. These structural similarities suggest that our simulations capture not only the statistical features of rupture but also reproduce key microscopic geometries observed experimentally.

We compared the evolution of electronic transmission during the simulated junction pulling for the three potentials. The NEP model reproduced subtle experimental features, such as parity oscillations in individual transmission traces and a prominent 1 G_0_ peak in the 1D-histogram. While the EAM potential also reproduced some experimental features, including distinct peaks in the 1D-histogram, it is ill-suited for simulations with molecules. Transmission traces from the ReaxFF potential did not resemble those observed experimentally. Although the 1D-histogram showed distinct peaks at integer values of G_0_, caution is advised when using this potential to simulate Au–Au break junctions. Finally, we compared the predicted thermopower distributions for each potential at each pulling speed. Both the NEP model and the EAM potential reproduced the experimentally observed thermopower of around −1 μV/K for few-atom gold junctions. However, as discussed above, the EAM potential is not easily applicable to systems containing molecules. The thermopower distributions obtained from the ReaxFF potential did not resemble the experimentally observed distributions, likely due to the unusual junction geometries that formed during the pulling simulations.

Future studies could use the NEP model to generate accurate, slow-pulled MD simulations of break junctions in combination with other deep learning methods. For example, ML can substantially reduce the computational effort of calculating the electronic conductance.^62–65^ This would enable more detailed investigations into the dynamics of the electronic transmission that is not possible with the limited sample sizes of our study. It would also be interesting to investigate temperature effects on pulling-speed dependence and other observables. At present, we trained and validated the NEP model on room-temperature data to later target room-temperature molecular junctions. An extension to cryogenic conditions (e.g., 4.2 K) in addition to the room-temperature data was deemed beyond the scope of the present study, but we plan to pursue this direction in future work.

A recent study demonstrated that a NEP model trained on 16 elemental metals and their alloys performs well across a range of systems.^66^ Although it may underperform in the amorphous interelectrode region, such a general model can be fine-tuned to improve performance, further reducing training costs and broadening applicability. However, increasing model complexity comes at the expense of computational speed. The trade-off between accuracy and evaluation speed must therefore be assessed on a case-by-case basis. With the performance of the NEP model, future studies could also look at other properties such as shot noise or thermal conductance. Other important metrics that could be looked at and compared with experimental results include thermal transport^67–69^ and shot noise.^70,71^ It would also be interesting to calculate how these experimental observables change with temperature.

In conclusion, this study contributes to the body of knowledge on the electronic properties of Au–Au junctions, highlighting the importance of using accurate and efficient computational models in deciphering the underlying physics of nanoscale electronic transport. By demonstrating the effectiveness of the NEP model, we pave the way for future research aimed at extending its applicability to more complex systems, including break junctions with molecules incorporated. With the advancements in modern computing and ML, we are steadily bridging the gap between experimental observations and in silico simulations.

## Methods

### Machine Learning Potential

Among various MLPs available, we chose NEP, a feed-forward neural network model trained using a separable natural evolution strategy.^72^ NEP has been shown to have excellent performance for both crystalline and amorphous inorganic materials in terms of both accuracy and computational efficiency due to its native GPU implementation.^33,73–77^ In Section 1 of the Supporting Information, we provide a brief description of the NEP approach. We refer to the original publications for the full derivation.^33^ The NEP model takes as input the structures and is trained to reproduce the energies of each structure and the forces on each atom of each structure in the training set. The chosen hyperparameters of the NEP model are shown in Table S1.

For the training data generation, we use the DFT Python code GPAW,^78^ within the Atomic Simulation Environment.^79^ We use the Perdew–Burke–Ernzerhof (PBE) exchange–correlation functional^80^ and a double-ζ polarized (DZP) basis set on all atoms. Dispersion corrections were included using the DFT-D3 method with Becke–Johnson damping.^81,82^ We employ PBE with a DZP basis, which has been shown to yield reasonable results for Au–Au and Au–molecule junctions.^83–87^ As we are not looking at thermochemistry, we are less concerned with inaccuracies in energies.^88^ In addition, as we show evidence of in Figure 2, our choice of computational level shows excellent agreement with experimental measurements for dispersion relations of bulk Au. The Kohn–Sham equations were solved self-consistently until the change in energy per valence electron was below 10^–5^ eV and the change in density was less than 10^–5^ electrons per valence electron. We used only the Γ-point for **k**-space sampling due to the size of the central region (6 × 6 Au electrodes). We show a comparison of the forces from a calculation at the Γ-point with a calculation using a 2 × 2 × 1 **k**-point space in Figure S4. The error on the forces for the Γ-point calculation is negligible.

We used an active learning loop to generate the training data for the NEP model. Although the NEP model was the final model used for production simulations, we employed a surrogate model—performant implementation of atomic cluster expansion (PACE)^89^—during the data acquisition stage. This choice was motivated by practical considerations: PACE provides a built-in D-optimality criterion to assess model uncertainty^90^ and is compatible with LAMMPS, the software used for performing the MD simulations, allowing efficient on-the-fly sampling. The D-optimality criterion estimates how much the model is extrapolating on new samples and flags samples that require significant extrapolation for further analysis. Importantly, the NEP model was never trained on outputs from PACE. Instead, NEP was trained exclusively on DFT-calculated energies and forces. PACE was used only to guide the selection of new data points during active learning. Training NEP directly within the active learning loop would have been computationally inefficient due to NEP’s longer training time and lack of integrated uncertainty estimation. By using PACE as a fast, uncertainty-aware proxy, we could efficiently explore configuration space and identify relevant structures for DFT labeling. Once the active learning loop produced a sufficiently diverse and well-covered training set, we trained the final NEP model and used it for all subsequent production simulations.

The training set was generated using systems composed of 6 × 6 Au electrode with one or two 4,4′-bipyridines placed between them. These molecules were included such that the trained MLP could be used for further work to simulate junctions with 4,4′-bipyridine. For the NEP model to describe the system with 4,4′-bipyridines well, it also needs to describe the gold well. First, we seeded the training set with randomly selected structures from an ab initio MD calculation of a 4,4′-bipyridine system with 6 × 6 Au-electrodes that was initiated at 700 K and in the microcanonical ensemble (*NVE*). Hydrogen mass repartitioning was used to facilitate larger timesteps, setting the time step at 1 fs.^91^ Mass repartitioning works by increasing the mass of hydrogens and correspondingly reducing the mass of the heavy atoms where the hydrogens are connected. Thus, the total mass of the molecule is kept the same.

For the pulling simulations used in the active learning, we applied a pulling speed of 2 × 10^–4^ Å/fs using timesteps of 0.5 fs. The temperature was maintained at 300 K in the *NVT* ensemble, controlled by stochastic velocity rescaling with a temperature damping parameter of 100 fs.^92^ Using a global thermostat during pulling is important as it allows the kinetic distribution in the various parts of the system to remain inhomogeneous while avoiding overheating the whole system due to the mechanical work.

The active learning procedure went as follows (see also Figure S1):

1. Initial training: Train a PACE model on the currently available training data.
2. MD simulation and data selection: Run MD trajectories using the PACE model until the trajectory either ran to completion or crashed. During these trajectories, mark snapshots in which the predictions of the model were insufficient, based on the D-optimality criterion.
3. Force calculations: Calculate the forces and energies using DFT on all marked snapshots. These structures alongside their forces and energies are then added to the current training set, and the loop starts over at step 1.
4. Loop termination: Terminate the active learning loop when a full MD run is complete without any structures marked as poorly described.

We compare our NEP model with two other classical potentials frequently used to investigate Au–Au junctions and junctions with small molecules: the EAM potential^34^ and ReaxFF.^35^ We use the EAM potential parameters provided by Sheng et al.^93^ and the ReaxFF parameters provided by Bae and Aikens.^94^ The parameter set of the ReaxFF potential improves upon a previous parameter set optimized for Au–S–C–H systems.^95^ Both the improved parameter set and its predecessor have been used in prior studies for simulating Au–Au junctions interfacing with small organic molecules.^64,96,97^

### Nonequilibrium Molecular Dynamics

In our study, we modeled a break junction setup by placing two layers of a 10 × 10 fcc(111) Au on either side of a short wire, totaling 717 Au atoms (Figure S2). Hundreds of MD simulations (see Table 2) were started from the same geometry-optimized configuration with velocities generated with different random distributions. As Au has a large atomic mass, we used a time step of 5 fs. As in the MD simulations used to generate the training set, the temperature was controlled by stochastic velocity rescaling with a damping parameter of 100 fs.^92^

**Table 2:** MD Parameters of the Simulated Pulling Experiments

method	pulling speed (Å/fs)	dump interval (every × Å)	final steps	#runs
NEP	10^–5^	1.28 × 10^–3^	10k	512
NEP	10^–6^	2.56 × 10^–4^	100k	512
NEP	10^–7^	2.56 × 10^–5^	100k	512
NEP	10^–8^	3.00 × 10^–5^	100k	100
NEP	10^–9^	4.00 × 10^–5^	150k	22
EAM	10^–5^	1.28 × 10^–3^	10k	512
EAM	10^–6^	2.56 × 10^–4^	100k	512
EAM	10^–7^	2.56 × 10^–5^	100k	512
ReaxFF	10^–5^	1.28 × 10^–3^	10k	128
ReaxFF	10^–6^	2.56 × 10^–4^	100k	128

Periodic boundary conditions were imposed in the plane of the electrodes (*x*- and *y*-directions), with nonperiodic, shrink-wrapped boundary conditions along the pulling axis (*z*-direction). Shrink-wrapped boundary conditions allow the boundary in the *z*-direction to adjust, positioning itself to encompass all atoms in this dimension as the atoms are pulled.^64,98^ In all simulations, the two outermost layers of gold were constrained to their ideal lattice positions. As the amount of time required to run a full simulation increases when the pulling speed is decreased, we run a decreasing number of trajectories. Table 2 details the number of trajectories computed for each pulling speed and force field employed.

Hereafter, pulling speeds are expressed in Å/fs, aligning with MD simulation conventions, rather than the nm/s typically used in experimental studies. For ease of comparison, we note that our slowest pulling speed is 10^–9^ Å/fs, which corresponds to 10^5^ nm/s—that is, the simulations pull 4 orders of magnitude faster than typical experiments and 2 orders of magnitude faster than the fastest experiment performed at 1000 nm/s. All MD simulations were performed using LAMMPS (28 Mar 2023-Development).^98,99^

Each MD simulation started with a short equilibration run of 5 × 10^3^ timesteps where no pulling was performed. In Figure S6, we include a 1D-histogram of breaking distances at a pulling speed of 10^–6^ Å/fs where we increase the amount of initial equilibration timesteps from 5 × 10^3^ to 5 × 10^5^. The average breaking distance increases slightly (∼1 Å), but the distributions are wide so this increase is likely within the statistical variance of the simulations.

Subsequently, we started pulling the left electrode at a fixed velocity. As the Au junctions (and MD calculations in general) break stochastically, multiple trajectories were simulated at each pulling speed, each with a unique starting seed. We dump trajectory snapshots during the simulation, adjusting dump frequency according to the pulling speed as detailed in Table 2.

Instead of pulling for a fixed number of timesteps, we periodically check whether the Au–Au junction has ruptured. This is done by creating an adjacency matrix of the junction from the covalent radii of Au atoms. The adjacency matrix can be thought of as a graph that can be periodically checked whether it is disjoint. A disjoint graph means that a rupture of the junction has occurred. After a rupture is registered, the simulation continues briefly before termination; the specific duration for each pulling speed is outlined in Table 2 in the “Final steps” column.

Although our break junction model includes just a few hundred atoms, its size is representative of the tips of the contacts that dictate the experimental response. And, as we will show, this minimal system likely retains most of the physically relevant behavior. First, our simulations reproduce the well-known single-atom point contact—and even atom-chain formation—seen in experiments. Second, phonon dispersion calculations on an 8 × 8 × 8 Au electrode (Figure 2) align almost perfectly with the measured spectrum, demonstrating that larger electrodes add no further long-range effects.

### Electronic Transport Calculations

We calculate the electronic transmission using the tbtrans module of SIESTA.^100,101^ tbtrans uses the nonequilibrium Green’s function method to calculate the Landauer transmission. In these calculations, we use the PBE functional^80^ with a single-ζ basis set, as the computational costs of a more complete basis set for such a large system would be unaffordable. We calculate the electronic transmission at ±0.05 eV of the Fermi level and take the average between those two data points. Furthermore, we calculate the thermopower using the following equation^102^

$$S\left(T,E_{F}\right)\approx \frac{\partial T\left(E\right)}{\partial E}{|}_{E=E_{F}}\frac{\left\langle {\left(E-E_{F}\right)}^{2}\right\rangle }{T\left(E_{F}\right)eT}=\frac{k_{B}^{2}\pi^{2}T}{3e}\frac{\partial \ln T\left(E\right)}{\partial E}{|}_{E=E_{F}}$$ 1

here, *T* represents the temperature, *E*_F_ is the Fermi energy, *E* is the energy, *e* is the elementary charge, and *k*_B_ is the Boltzmann constant.

## Supplementary Material



## Data Availability

In- and output files for exemplary MD trajectories are available at 10.17894/ucph.9fa575-cd-7854-435a-aa90-412fcd1dc57e and a few randomly selected traces can be visualized at 10.19061/iochem-bd-9-11. An example folder of a transmission input file is uploaded at 10.17894/ucph.fb3a18ed-7e45-4c8b-8ff7-43ab81834bb4. A version of this manuscript has been uploaded to Chemrxiv.^103^

## Notes

NEP model mathematical description; NEP model parameters; training workflow; starting junction geometry; optimized unit-cell parameters; *k*-point comparison of forces; test set performance; test of breaking distance versus equilibration timesteps; comparison of EAM and ReaxFF breaking structures; parity oscillations; thermopower of gold junctions; push–pull experiment with 4,4′-bipyridine; pulling with a spring constant; individual pulling force traces with NEP; breaking force analysis; fitting multiple peaks; push–pull of Au junctions; 1D-histograms of forces (PDF)
