# Supplementary material for: Bridging the Gap: Using Machine Learning Force Fields to Simulate Gold Break Junctions at Pulling Speeds Closer to Experiments
Source: ACS Nano. 2025 Nov 13;19(46):39735–46. doi: 10.1021/acsnano.5c11887 (PMC12674199; doi:10.1021/acsnano.5c11887)
Supplement: Supplementary file 1 [file nn5c11887_si_001.pdf]

Supporting Information: Bridging the Gap: Using Machine Learning Force Fields to Simulate Gold Break Junctions at Pulling Speeds Closer to Experiments

William Bro-Jørgensen,† Joseph M. Hamill,† Davide Donadio,‡ and Gemma C. Solomon*,†,¶

†*Department of Chemistry and Nano-Science Center, University of Copenhagen, Universitetsparken 5, DK-2100, Copenhagen Ø, Denmark*

‡*Department of Chemistry, University of California, Davis, Davis, California 95616, United States*

¶*NNF Quantum Computing Programme, Niels Bohr Institute, University of Copenhagen, Denmark*

E-mail: gsolomon@chem.ku.dk

1 NEP model mathematical description

The descriptor for atom i consists of radial and angular components. The radial descriptors are defined as

$$q_n^i = \sum_{j \neq i} g_n(r_{ij}) \quad \text{with} \quad 0 \leq n \leq n_{max}^R. \tag{1}$$

Here, n_{max}^R is a hyperparameter that sets the number of radial descriptor components, and r_{ij} is the interatomic distance between atom i and atom j. The summation runs over all the

neighbors of atom i within a given cutoff radius. In NEP, each $g_n(r_{ij})$ is defined as a linear combination of $N_{bas}^R + 1$ basis functions:

$$g_n(r_{ij}) = \sum_{k=0}^{N_{bas}^R} c_{nk}^{ij} f_k(r_{ij}) \tag{2}$$

with

$$f_k(r_{ij}) = \frac{1}{2}\left[T_k\left(2\left(\frac{r_{ij}}{r_c^R} - 1\right)^2 - 1\right) + 1\right] f_{cut}(r_{ij}). \tag{3}$$

Here, N_{bas}^R is another hyperparameter that controls the amount of basis functions used to build the radial descriptor functions, $T_k(x)$ is the kth order Chebyshev polynomial of the first kind and $f_{cut}(r_{ij})$ is a cutoff function defined as:

$$f_{cut}(r_{ij}) = \begin{cases} \frac{1}{2}\left[1 + \left(\cos \pi \frac{r_{ij}}{r_c^R}\right)\right], & r_{ij} < r_c^R \\ 0, & r_{ij} > r_c^R. \end{cases} \tag{4}$$

Here, r_c^R is the cutoff distance of the radial descriptor components and is a hyperparameter that can be tuned. The angular descriptor consists of 3-body to 5-body terms and is defined as

$$q_{nl}^i = \frac{2l+1}{4\pi} \sum_{j \neq i} \sum_{k \neq i} g_n(r_{ij}) g_n(r_{ik}) P_l\left(\cos \theta_{ijk}\right). \tag{5}$$

In this equation, $P_l\left(\cos \theta_{ijk}\right)$ is the Legendre polynomial of order l, while θ_{ijk} is the angle formed by the ij and ik bonds. The radial functions $g_n(r_{ij})$ and $g_n(r_{ik})$ take the same form as Equation 2 though they can have different cutoff distances and basis sizes, and are controlled by the hyperparameters r_c^A and N_{bas}^A, respectively. The direct numerical evaluation of Equation 5 is challenging. Consequently, it can be reformulated to address these concerns. Readers are referred to Fan et al.,[S1] where the reformulation and the explicit 3-, 4-, and 5-body terms are outlined.

2 NEP model parameters

In Table S1, we show the hyperparameters chosen for our NEP model and the training setup. All other settings have been kept as their default values as defined in the `GPUMD` program.

Table S1: Hyperparameters for the NEP model.

Hyperparameter	Value
NEP Version	4
Atomic species	H C N Au
r_c^R (Å)	8
r_c^A (Å)	4
N_{bas}^R	12
N_{bas}^A	12
n_{max}^R	10
n_{max}^A	6
l_{3b}^{max}-l_{4b}^{max}-l_{5b}^{max}	4-2-1
N_{neu}	40
Batch size	1024
N_{gen}	4×10^6

In Table S1, n_{max}^A is the size of the angular basis, l_{3b}^{max}-l_{4b}^{max}-l_{5b}^{max} is the expansion order for the angular terms, N_{neu} is the number of neurons in the hidden layer, batch size is the batch size used for training, and N_{gen} is the number of generations used by the separable natural evolution strategy algorithm that is used to train the force field.

3 Training workflow

We show an illustration of the training workflow in Figure S1. A detailed description of the training is provided in the Methodology of the manuscript.

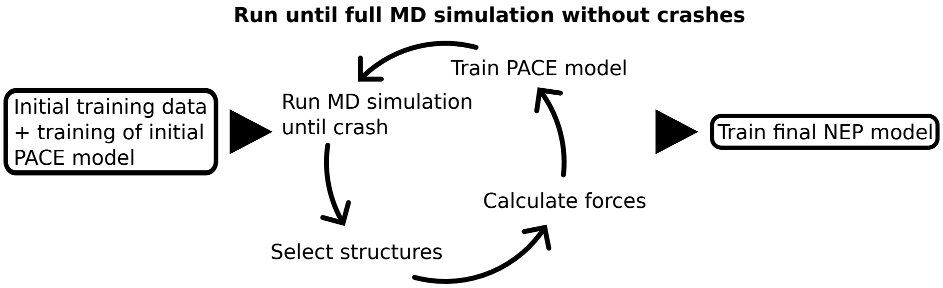

Figure S1: Illustration of the workflows to train the NEP model.

4 Starting junction geometry

We show the initial junction geometry in Figure S2

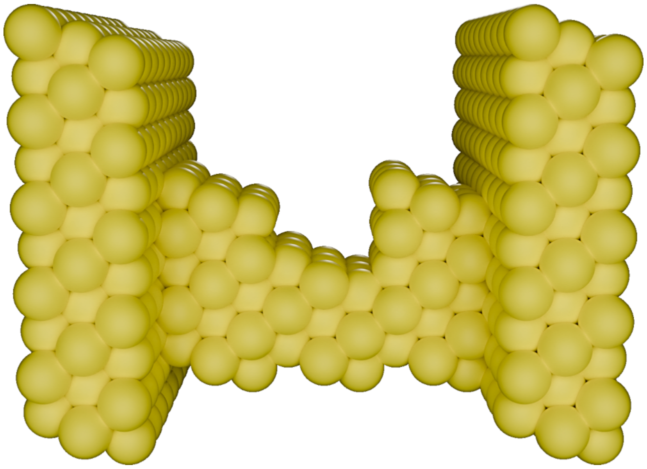

Figure S2: Starting junction setup for the MD calculations. The two outermost layers on both sides are frozen and the rest of the Au atoms are allowed to move freely in the MD calculations.

5 Optimized unit-cell parameters

In Figure S3, we show the energy-volume curves for bulk $8 \times 8 \times 8$ Au calculated with density functional theory (DFT) (blue circles) and the three potentials: the NEP model (orange circles), the EAM potential (green circles), and the ReaxFF potential (red circles). The lines are fits from Equation 6[S2]

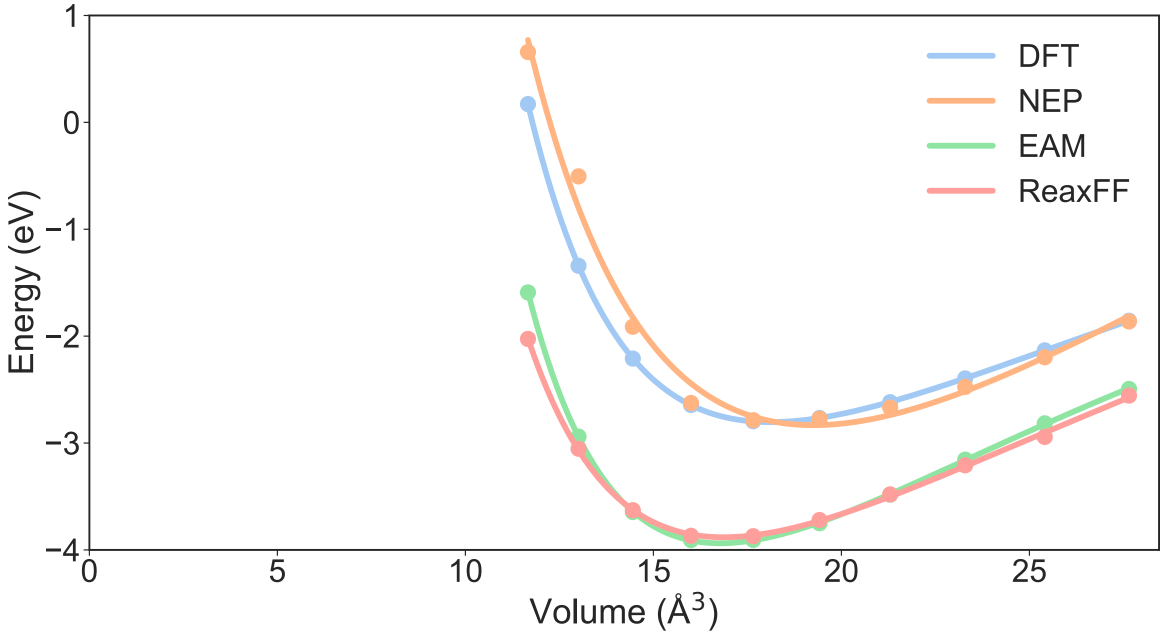

Figure S3: Energy-volume curves for bulk fcc Au calculated using DFT (blue circles), NEP (orange circles), EAM (green circles), and ReaxFF (red circles). The lines of the same color are fitted to Equation 6.

$$E(V) = E_0 + \frac{9V_0B_0}{16}\left\{\left[\left(\frac{V_0}{V}\right)^{2/3} - 1\right]^3 B_0' + \left[\left(\frac{V_0}{V}\right)^{2/3} - 1\right]^2 \left[6 - 4\left(\frac{V_0}{V}\right)^{2/3}\right]\right\} \quad (6)$$

From Equation 6, we can get the lattice constants for each force field and the bond lengths between Au atoms in an fcc(111) unit cell. We show a comparison of these in Table S2.

Table S2: Optimized unit-cell parameters for all force fields.

Force field	Au-Au bond distance (Å)	Lattice constant (Å)
NEP	2.2628	4.255
EAM	2.0641	4.064
ReaxFF	2.0717	4.071
DFT	2.1682	4.165
Experiment[S3]	2.0655	4.065

6 k-point comparison of forces

In Figure S4, we compare the forces between a calculation with a **k**-point sampling of 1x1x1 (small) and a **k**-point sampling of 2x2x1 (big). As there is a high amount of overlapping points, the distribution of points have been colored via kernel density estimation. Each point in the dataset represent an individual component (x, y, or z) of a force vector. Overall, the error between the two calculations is small as indicated by the mean absolute deviation (MAD), root mean square deviation (RMSD), and maximum absolute error detailed in the legend of Figure S4A.

Figure S4C illustrates the error of the forces on each atom using a color gradient going from white to red, where a deeper red signifies a larger deviation. The error is quantified using the L2-norm, essentially measuring the magnitude of difference between the forces in all three spatial directions:

$$\sqrt{\sum_{i=1}^{3}(f_{DFT_{small},i} - f_{DFT_{big},i})^2}. \tag{7}$$

Here, the sum runs over each of the three directions (x-, y-, and z-direction), and $f_{DFT_{small}}$ and $f_{DFT_{big}}$ is the force calculated by the big or small DFT, respectively.

Overall, we see that the error on the forces between the big and small **k**-point sampling scheme is minimal.

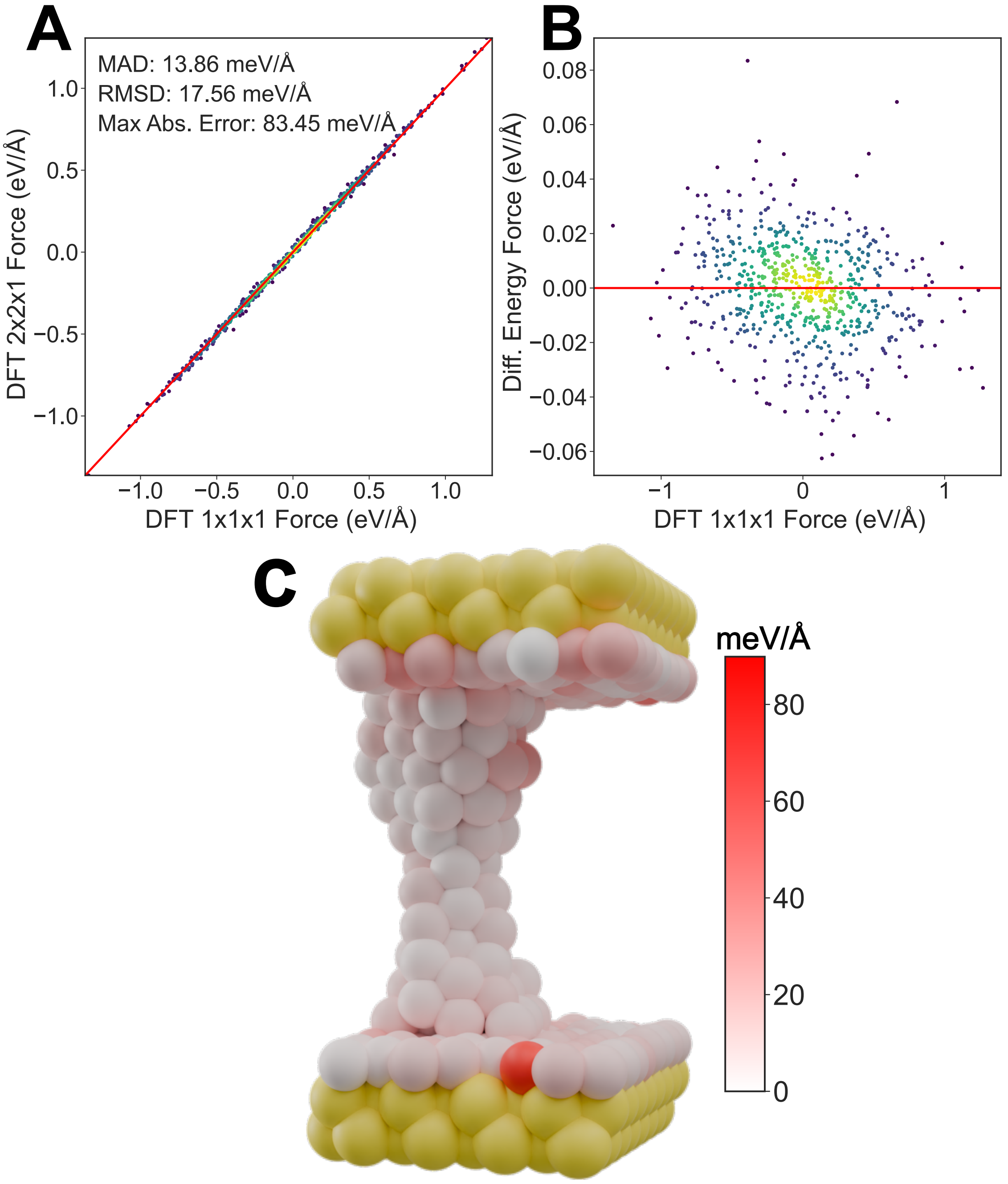

Figure S4: Comparison of forces from a calculation with a 1x1x1 **k**-point sampling to that of a calculation with a 2x2x1 **k**-point sampling. (A) Each x-, y-, and z-component from each calculation is plotted against each other. (B) Force components from the 1x1x1 calculation versus the error between the 1x1x1 and the 2x2x1 calculation. (C) The magnitude of the error between the two calculations is plotted on the reference structure. The redder an atom, the bigger the error.

7 Test set performance

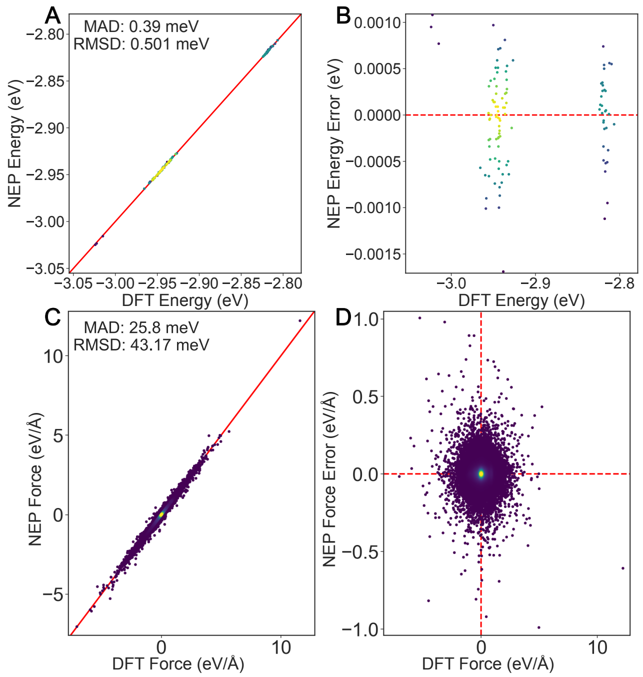

Figure S5: Training performance of NEP model at the final active loop iteration on the test set. (A) DFT energy versus the predicted NEP energies of each configuration. (B) Error on the energy estimate of each configuration vs. DFT energy per configuration. (C) DFT forces versus the NEP forces on each atom. (D) Error of the force estimate on each atom vs. DFT force components. The mean absolute deviation (MAD) and the root mean squared deviation (RMSD) is shown in A and C. The red solid and dashed lines act as guides to for the eye. Gaussian kernel density estimation have been used to color overlapping points.

We evaluate the accuracy of the NEP model on the test set from the final iteration of the active learning loop by comparing the energies (Figure S5A and B) and the Cartesian components of the forces (Figure S5C and D) of the test structures predicted by the NEP model with those calculated with DFT. Note that this test set also includes structures with 4,4'-bipyridine (44BPY) in the junction. As there is a large number of overlapping points, the points have been colored via Gaussian kernel density estimation – lighter color corresponds to higher density. The test set entails a broad range of energies and forces. The converged MAD and RMSD for the energies are 0.39 meV and 0.501 meV, respectively. For the forces, the MAD and RMSD are 25.8 meV/Å and 43.17 meV/Å, respectively. There are two regions of energies in Figure S5A and B. These are due to different sizes of unit cells and

different numbers of molecules in the central region in the structures in the test and training set. The error metrics suggest that the trained NEP model effectively replicates the forces calculated by DFT. Furthermore, as both training and test sets encompass a broad range of configurations that have been sampled from pulling molecular dynamics (MD) simulations, the resulting NEP model is stable and can be used reliably in nonequilibrium simulations of break junctions.

8 Test of breaking distance versus equilibration timesteps

In Figure S6, we show how the distribution of breaking distances changes depending on the number of timesteps we equilibrate for before we start pulling. In Table S3, we list the mean breaking distance and how many samples are simulated for each amount of equilibration timesteps. Though the mean breaking distance increases slightly when the equilibration is increased, it is to be expected. As we see for the original run (equilibration of 5×10^3 timesteps), the distribution of breaking distances is quite broad.

Table S3: Summary data for the runs.

Timesteps	Mean distance (Å)	# samples
5×10^3	12.0	512
5×10^4	12.2	32
5×10^5	13.2	32

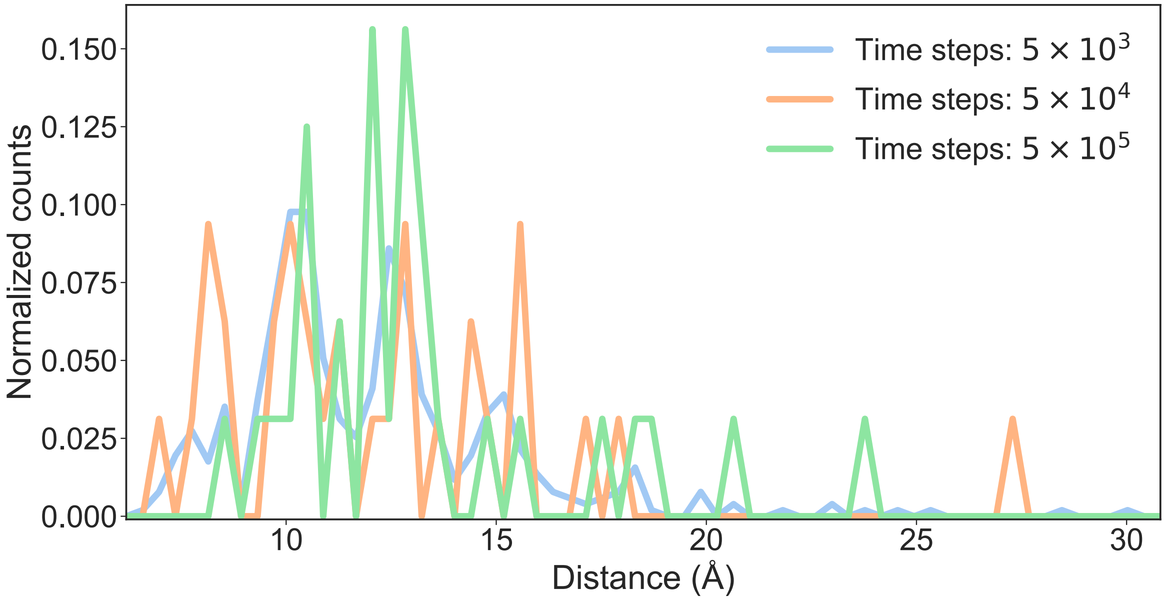

Figure S6: Distribution of breaking distance given an increasing amount of timesteps to equilibrate before pulling. The blue line is the distribution as shown in Figure 3 where we equilibrate for 5×10^3 timesteps, the orange line is for an equilibration of 5×10^4 timesteps, and the green line is for an equilibration of 5×10^5 timesteps.

9 Comparison of EAM and ReaxFF breaking structures

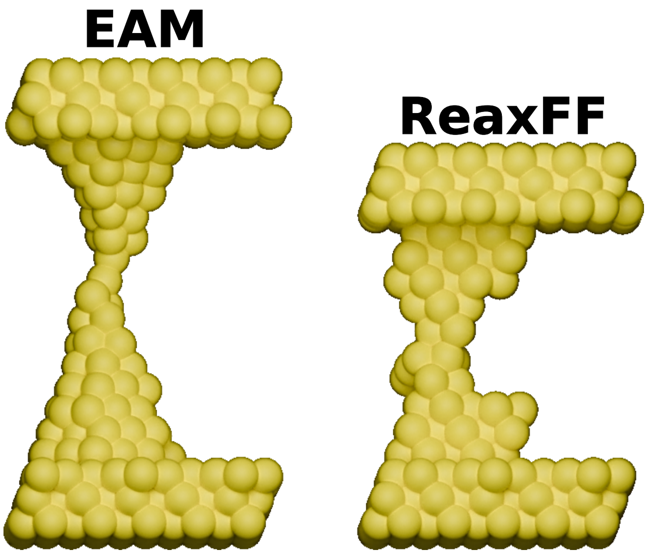

Figure S7: Illustration of an Au-Au junction just before breaking when using the EAM potential pulled at 10^{-7} Å/fs (left) and the ReaxFF potential pulled at 10^{-6} Å/fs (right).

In Figure S7, we compare two Au-Au junctions immediately before their rupture. The structure on the left is from a simulation using the EAM potential and the structure on the right is from a simulation using the ReaxFF potential. Notably, the central region of the ReaxFF-simulated structure closely resembles the fixed configuration of the Au atoms in the electrode areas. In contrast, the EAM-structure appears more amorphous. Additionally, while the Au atoms sitting directly on the electrodes in the EAM-simulated structure retain a crystalline form, they have reformed into a pyramid rather than maintaining the initial slab-like configuration. The qualitative shape of the EAM-simulated junction is similar to the structures that break at the shortest distance for the NEP model, as depicted in Figure 4 in the main manuscript.

10 Parity oscillations

In the main manuscript, we noted that the conductance traces exhibit parity oscillations consistent with prior experimental reports. In Figure S8, we compare the atomic configurations at a conductance just above 1 G_0 and immediately after the conductance falls to 1 G_0. The structure at slightly above 1 G_0 is characterized by a single gold adatom bridging two pyramidal electrodes, whereas the 1 G_0 plateau corresponds to a short, linear gold chain connecting the tips. Such gold structures with non-integer conductance states have been theoretically and experimentally explored previously.[S4]

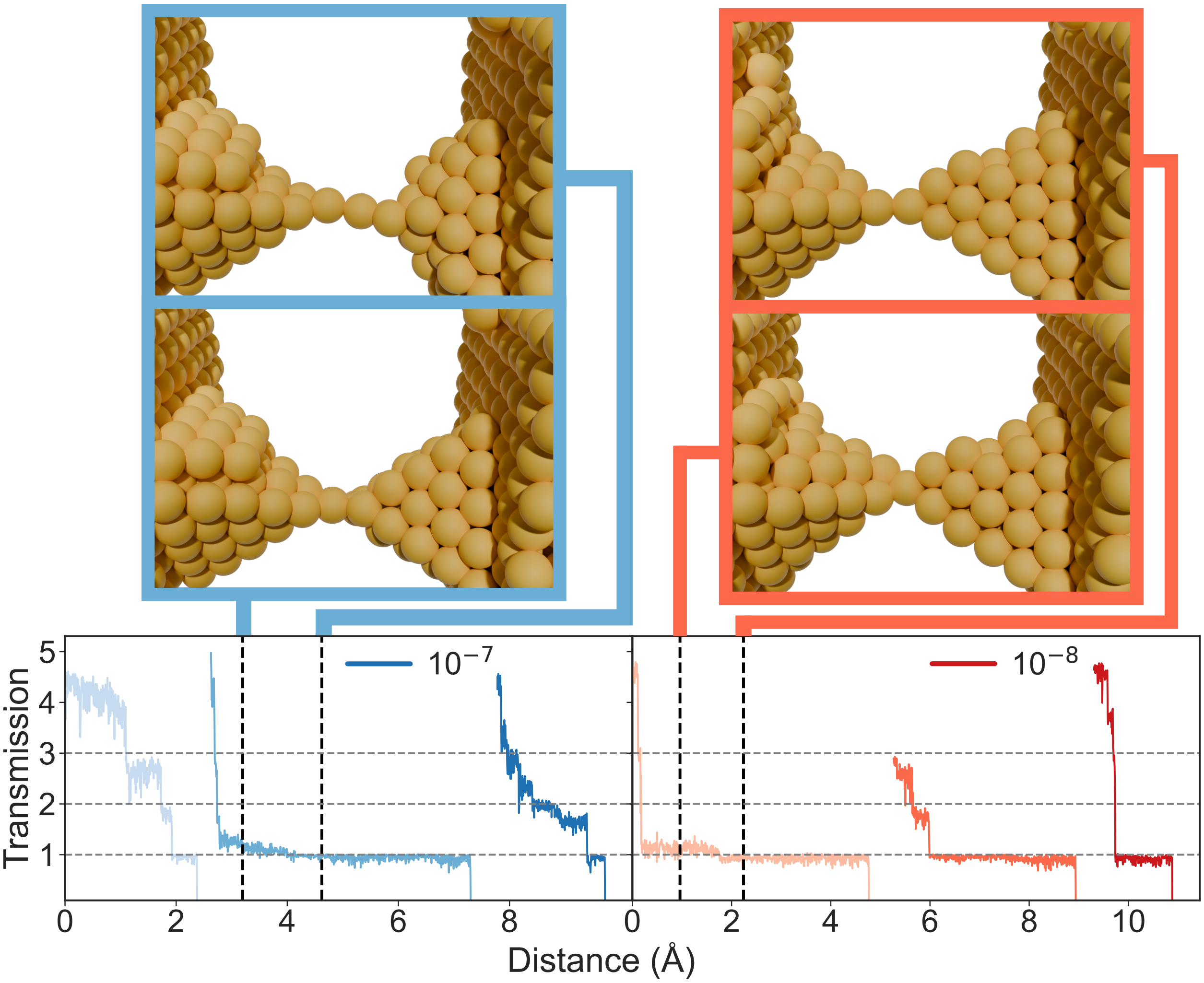

Figure S8: Visualization of illustrative structures leading to parity oscillations. Snapshots from the MD trajectory, stretched at a rate of 10^{-7} Å/fs (blue) and 10^{-8} Å/fs (red). Black, dashed vertical lines indicate the configurations captured in the top snapshots.

11 Thermopower of gold junctions

We plot the conductance versus thermopower for each potential at each pulling speed in Figure S9, Figure S10, and Figure S11.

Although it is challenging to conclude certainly due to the low amount of samples, it appears that the thermopower for the EAM potential and NEP model goes towards 0 μV/K at integer multiples of the conductance and then goes slightly negative in between. This is consistent with experiments measuring the thermopower of few-atom gold junctions.[S5,S6]

The data from the ReaxFF potential is at odds with both the experimental data and the data from the other two potentials. This is likely due to the unusual junction geometry that developed during the pulling simulation.

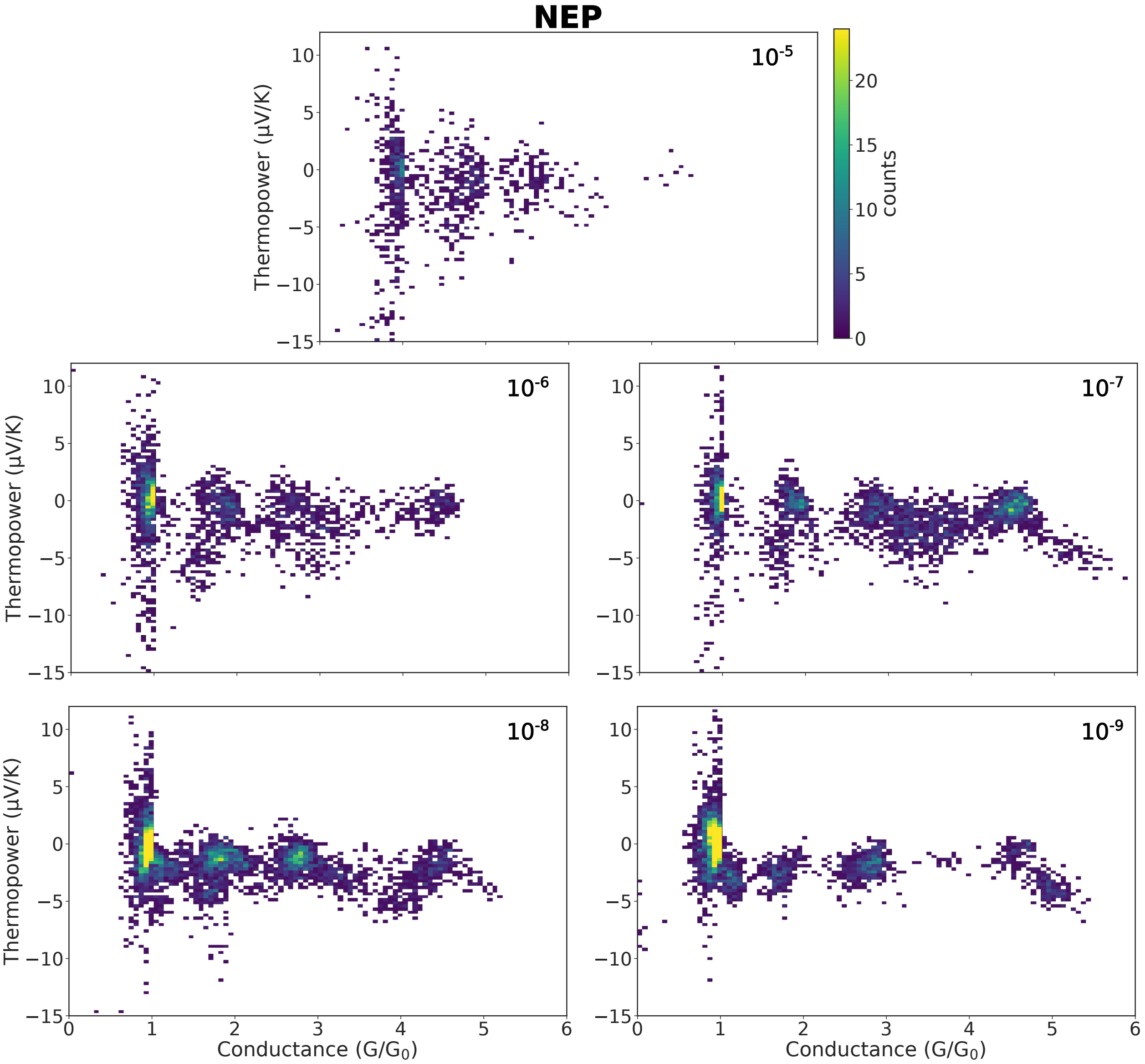

Figure S9: Conductance versus thermopower for the NEP model at each pulling speed.

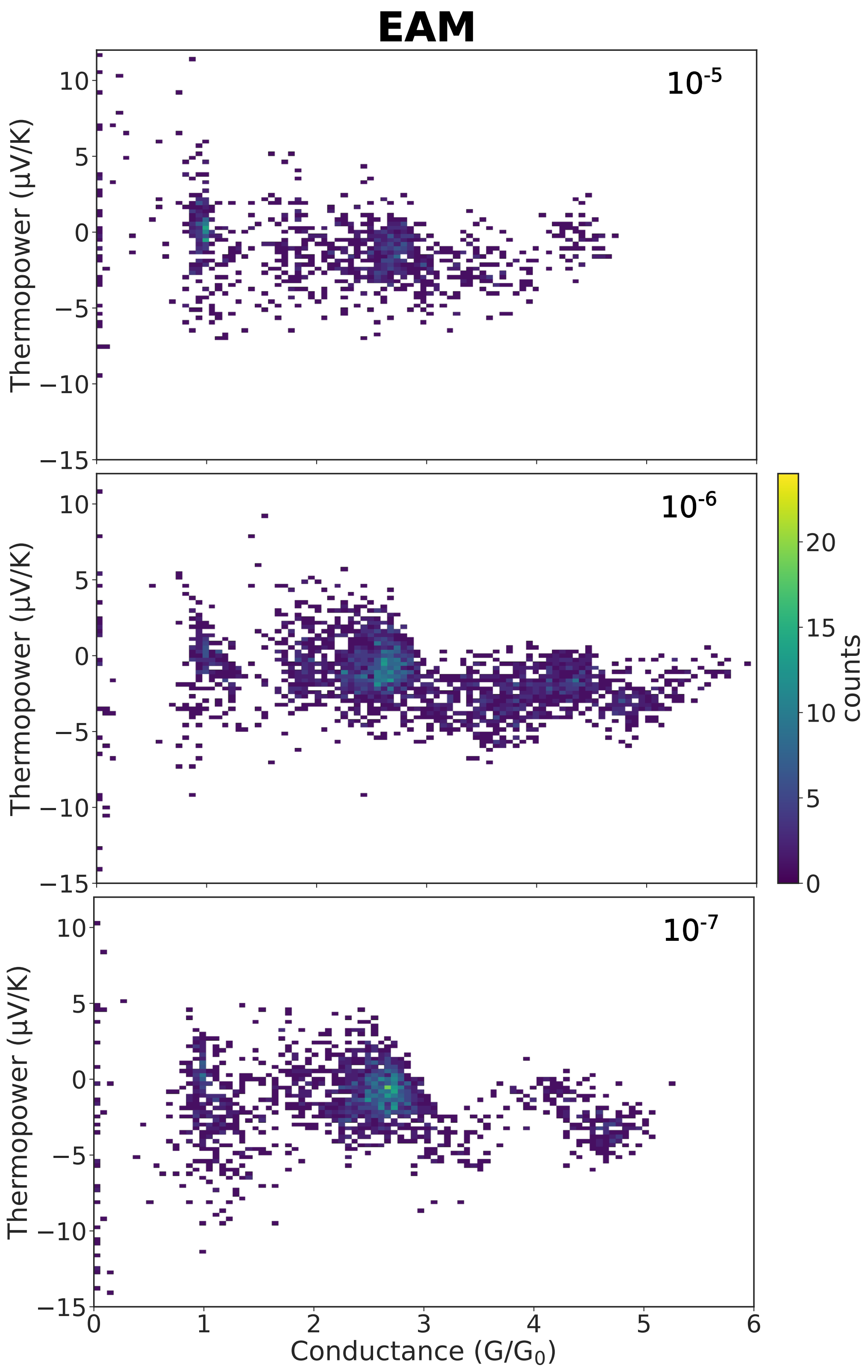

Figure S10: Conductance versus thermopower for the EAM potential at each pulling speed.

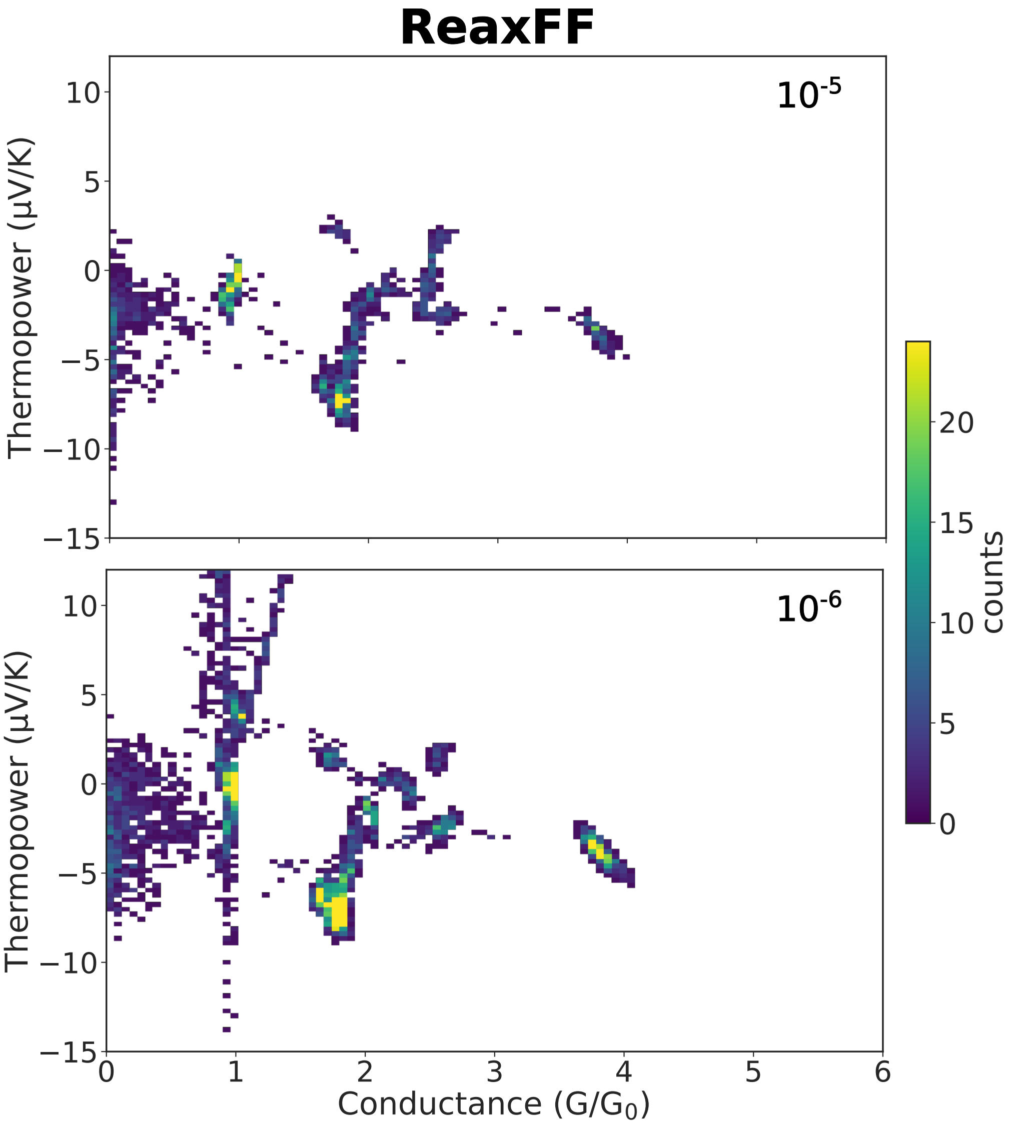

Figure S11: Conductance versus thermopower for the ReaxFF potential at each pulling speed.

12 Push-pull experiment with 4,4'-bipyridine

In Figure S12, we present the transmission data for the push phase of a push/pull junction simulation where we have included 44BPY. The junction was constructed in the same way as described in the manuscript. Afterwards, 44BPYs was scattered uniformly across the nanowire region and the structure was geometry-relaxed before further simulations. The junction was pulled apart as described in the main manuscript. The junction was pushed

back after the gold breaks. We plot the transmission (bottom of Figure S12) from the push phase of the trajectory and selected snapshots along the trajectory (top of Figure S12).

As 44BPY shifts from a fully stretched to a tilted conformation, its transmission increases smoothly with junction compression rather than jumping between distinct high- and low-conductance states. This continuous trend might imply that the commonly invoked mechanism for those discrete states may be more complex than originally thought.[S7] In experiments that also measure the conductance during the push phase, the change in conductance appears to happen to a larger extent.[S8,S9] We will examine these nuances in detail in a later publication.

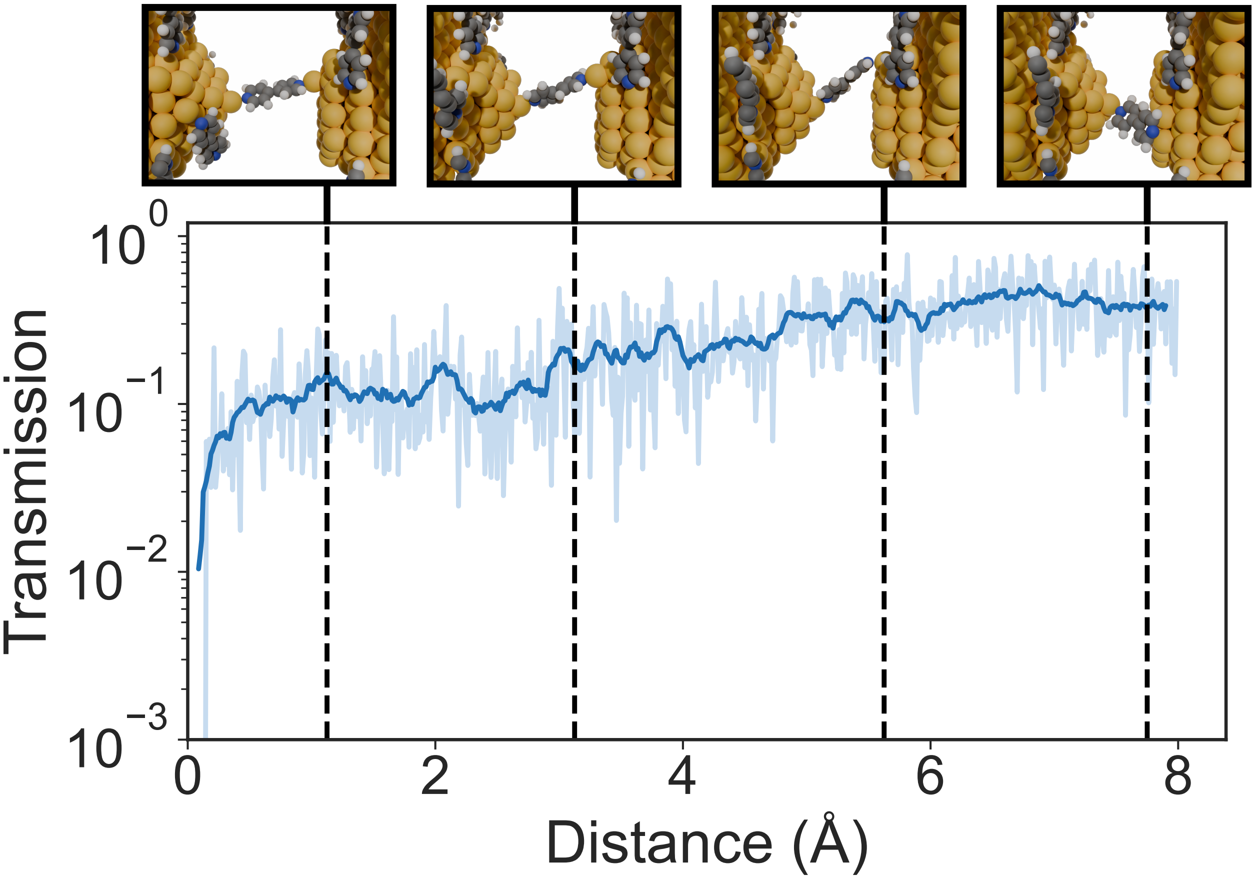

Figure S12: Visualization of the junction evolution and its impact on the conductance. (top) Snapshots from the MD trajectory, stretched at a rate of 10^{-8} Å/fs. (bottom) Conductance trace from the MD trajectory, where dashed lines indicate the configurations captured in the top snapshots. To show the general evolution of the transmission, we plot the actual transmission (shown in lighter hues) and its smoothed average (shown in darker hues).

13 Pulling with a spring constant

We performed pulling experiments using the steered MD module of LAMMPS. We applied a moving spring force to the bottom two layers of the right-most electrode. We used a timestep of 0.0075 ps. Periodic boundary conditions were imposed in the plane of the electrodes (x- and y-directions), with non-periodic, shrink-wrapped boundary conditions along the pulling axis (z-direction). Initial coordinates were read from a relaxed 10×10 fcc(111) gold slab with a short central wire (717 atoms total). Interactions were described by our NEP model. The MD simulations were started after an energy minimization (1×10^{-5} eV/Å convergence) and velocity initialization at 300K. Temperature was controlled on the electrodes only, using the same stochastic velocity rescaling thermostat as in the main manuscript.

We first equilibrated for 750 ps with both electrodes constrained to their initial positions by being tethered to a stationary spring with a very high force constant ($k = 10000$ eV/Å^2). After equilibration, the bottom tether was removed. Pulling was done with $k = 0.6242$ eV/Å^2. Atomic positions and forces were dumped periodically for analysis.

We plot the breaking distances of the Au-Au junctions under varying pulling speeds in Figure S13 to understand the junction evolution dynamics with a different type of pulling.

We first note that ruptures happen at multiples of approximately 2.6Å, slightly shorter than the Au-Au equilibrium distance in the bulk. This pattern of rupture at consistent intervals has been observed experimentally,[S10–S12] and the 2.6Å step is in agreement with recent measurements.[S13] As also seen in the main manuscript, the distinct peaks seem to blur at the slowest speed. This same blurring was also seen for the same speed in the main manuscript. Given the limited number of samples, this blurring could be a simple artifact from that. If we were able to run substantially more runs, a more clear peak structure might emerge.

As in the main manuscript, we observe a pronounced dependence of the breaking distance on pulling speed with the NEP model, suggesting that even at the lowest pulling speed the system is not in quasi-equilibrium, contrary to what might be inferred from simulations using

the EAM and ReaxFF potentials.

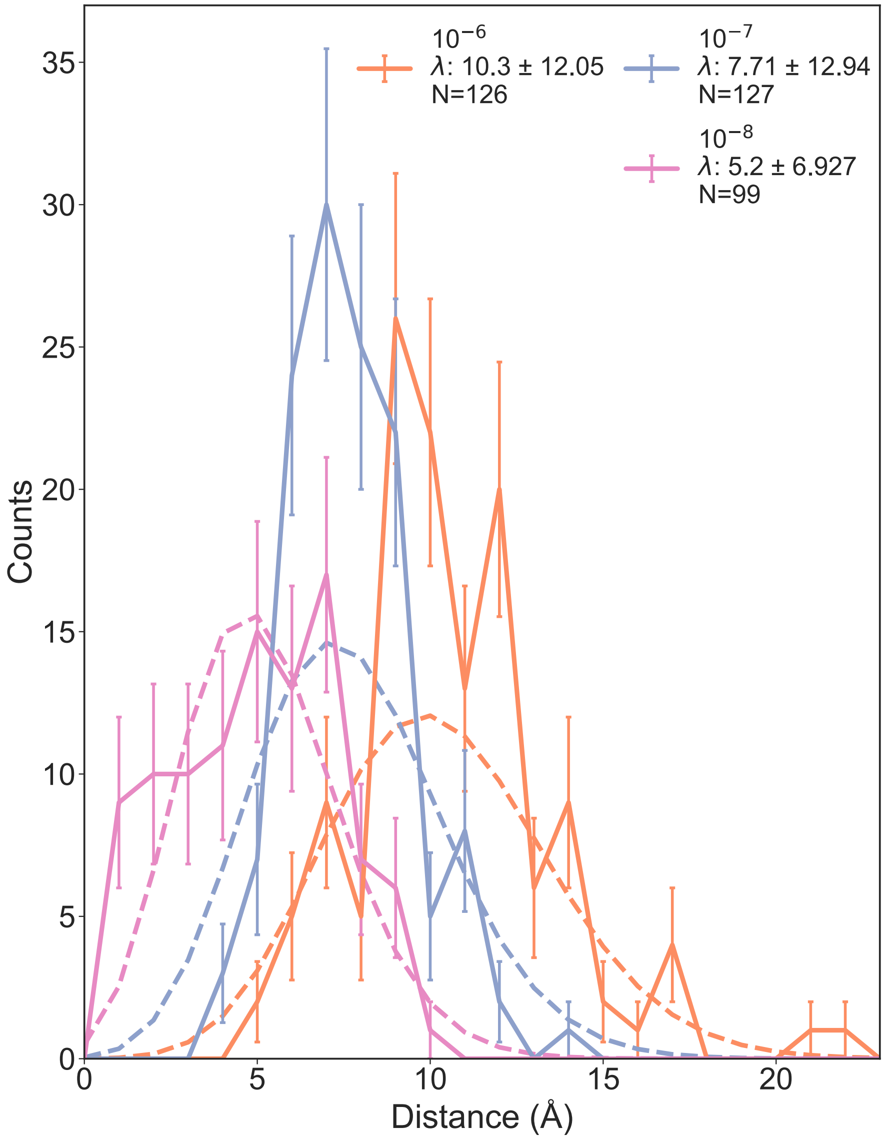

Figure S13: Breaking distance comparison for Au-Au junctions simulated at different pulling speeds with a spring constant of $k = 0.6242$ eV/Å^2 $\approx$ 10 N/m. The pulling speeds are 10^{-6} Å/fs (orange), 10^{-7} Å/fs (grey), and 10^{-8} Å/fs (pink). All simulations have been performed using the NEP model. Dashed lines represent Poisson fits with parameters λ (mean) indicated in the legend. The uncertainty of the counts in each bin is assumed to follow a Poisson distribution.

14 Individual pulling force traces with NEP

When using a harmonic spring, the total force along the pulling direction is recorded. Figure S14 shows two representative force-distance curves at each of the three pulling speeds.

All traces exhibit a pronounced initial force peak before the first plastic deformation, followed by a series of smaller slip events leading up to junction rupture. These slip events mirror experimental observations, where the force-distance curve has a steeper slope in the beginning and flattens as rupture approaches.[S14,S15] No baseline correction has been applied. For the slowest pull speed 10^{-8}, the simulation was run for 350 ns before data collection began.

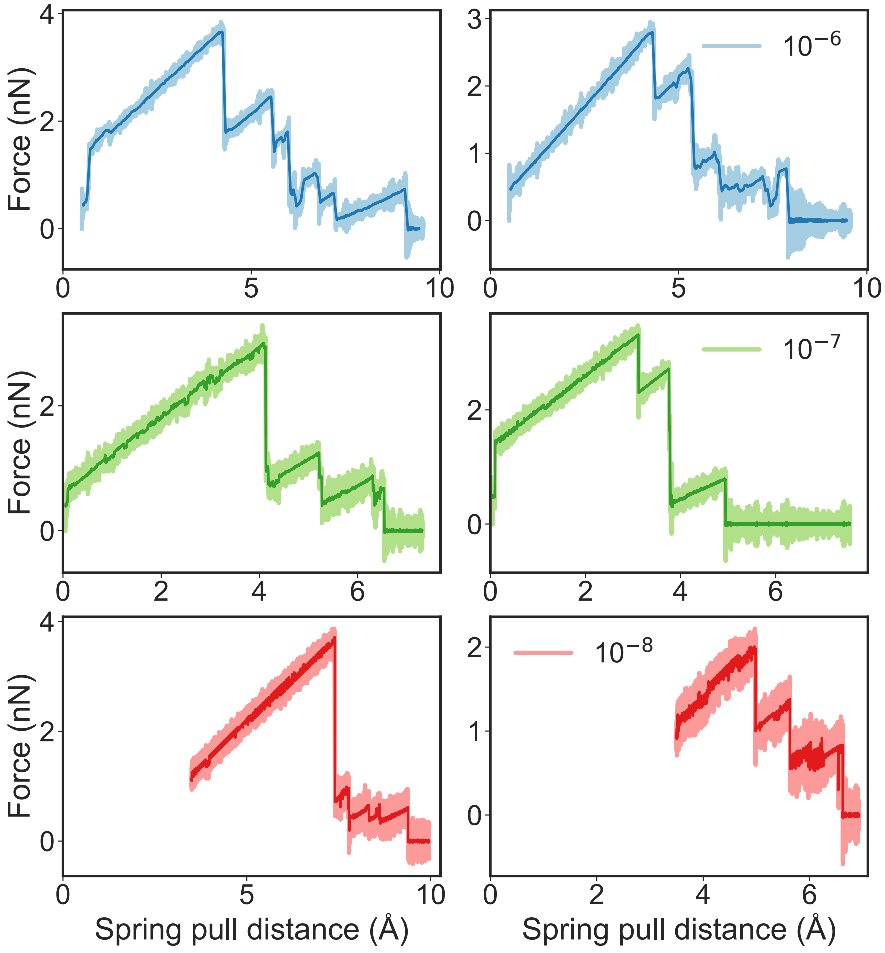

Figure S14: Two representative force-distance curves are shown for each pulling speed. The top, middle, and bottom rows correspond to pulling speeds of 10^{-6} Å/fs (blue), 10^{-7} Å/fs (green), and 10^{-8} Å/fs (red). In each plot, the lighter trace depicts the raw force data, while the darker trace denotes a rollingaverage. Note that the x-axis is the extension of the molecular spring, not the electrode separation.

15 Breaking force analysis

With access to the complete time evolution of the pulling force, we analyzed the force immediately prior to junction rupture. We adopted an approach similar to that of Pobelov et al.,[S16] but applied it to a larger junction configuration compared to their six-atom gold chain. Below, we briefly describe the procedure used to estimate the breaking force.

For each trace, we extracted the time-dependent force acting on the bottom electrode as reported by the LAMMPS `smd` module. The rupture point, t_{break}, was identified from

the force trajectory as described in the main text and is indicated by a dashed black line in Figure S15. Linear regression was performed on the force data from 5 ps before rupture up to t_{break}. The resulting fit function, $F_{fit}(t)$, was evaluated at t_{break} to determine the breaking force, $F_{bf} = F_{fit}(t_{break})$. The fit is shown as a solid black line in Figure S15.

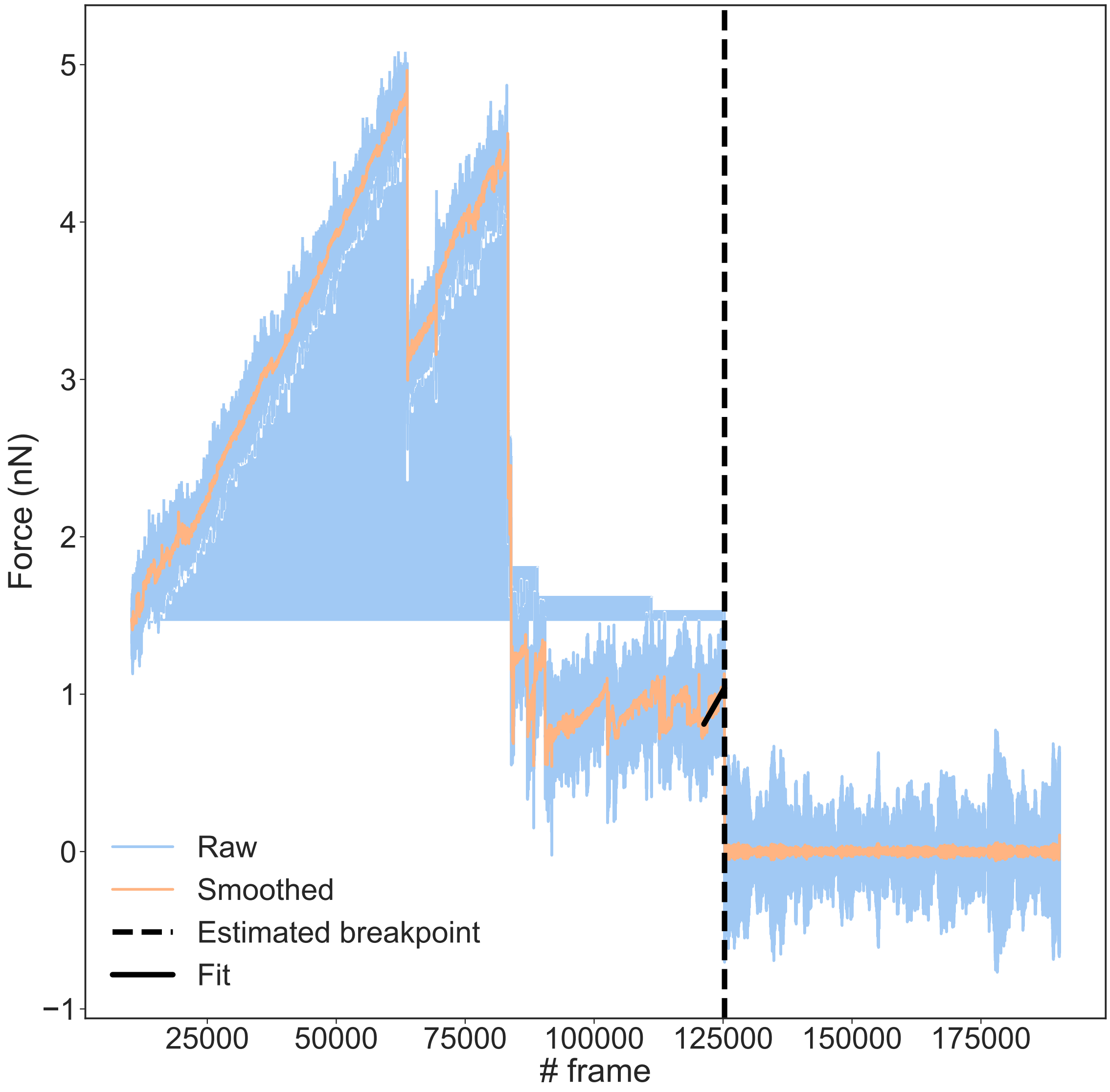

Figure S15: Illustration of the breaking force estimation. The raw force data is plotted in blue, and a smoothed version using a Savitzky-Golay filter is shown in orange. The estimated rupture point t_{break} is indicated by a dashed black line. The linear fit used to estimate the breaking force is shown as a solid black line.

We estimated the breaking force for each trace at each pulling speed and summarized the distributions in Figure S16A. A Gaussian was fitted to each distribution to extract the

average breaking force, with the fit parameters and uncertainties reported in the legend. To investigate the force dependence on the loading rate, we plotted the average breaking force as a function of loading rate, calculated by

$$r_F = k \cdot v. \tag{8}$$

Here, k is the spring constant (see "Pulling with a spring constant") and v is the pulling speed. These average breaking forces are shown in Figure S16B. A linear fit is included to guide the eye. As seen in Figure S16B, the breaking force has not yet plateaued, suggesting that we have not reached a regime of spontaneous rupture.

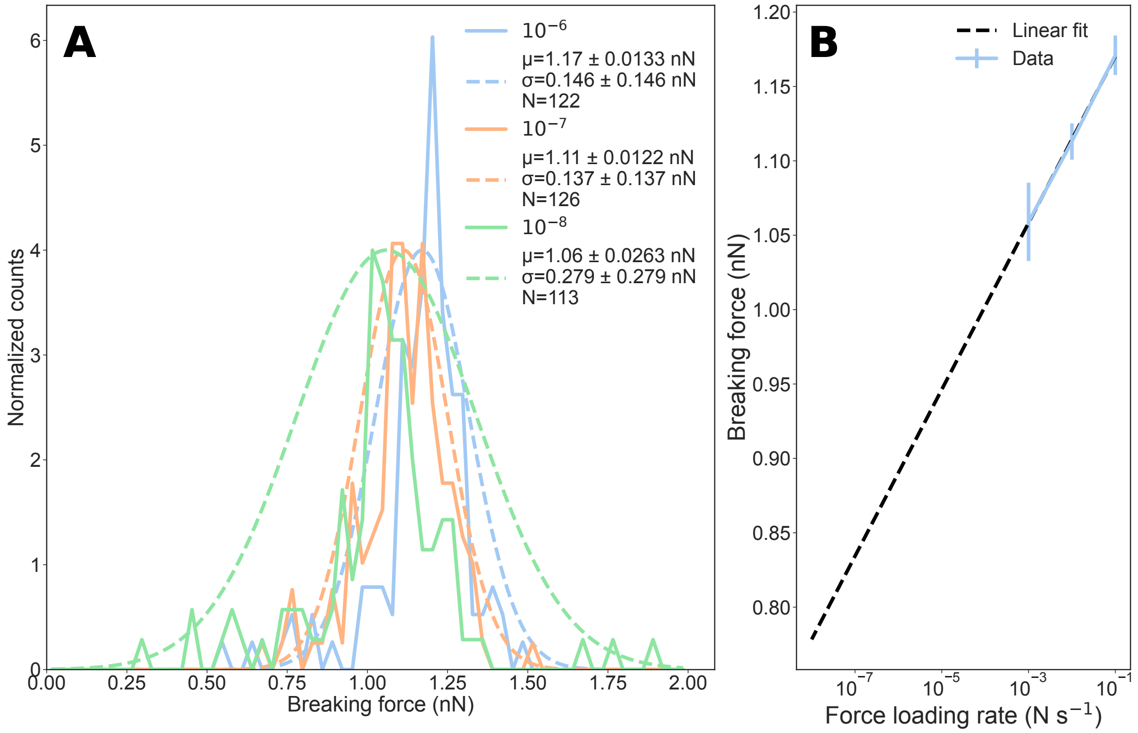

Figure S16: Dependence of breaking force on pulling speed. (A) Distributions of the force required to break the junction just prior to rupture at three different speeds: 10^{-6} Å/fs (blue), 10^{-7} Å/fs (orange), and 10^{-8} Å/fs (green). The dashed lines are Gaussian fits. The fitted parameters are shown in the legend along with their errors. (B) Plot of the mean from fitted Gaussians in (A). The error estimates are the error from the estimated mean of the Gaussian.

16 Fitting multiple peaks

As there are multiple peaks in the breaking distance plots shown in Figure 3, we attempt a fit with multiple Gaussians or Poisson distributions. For illustration purposes, we only do it for the 10^{-6} Å/fs dataset.

We fit multiple Gaussians or Poisson distributions according to Equation 9 and Equation 10, respectively:

$$f(x) = \Sigma_{i=1}^{3} A_i e^{-((x-\mu_i)^2)/(2*\sigma_i^2)} \quad (9)$$

$$f(x) = \Sigma_{i=1}^{3} e^{-\mu_i} \frac{\mu_i^x}{x!} \quad (10)$$

where $x = k - loc$.

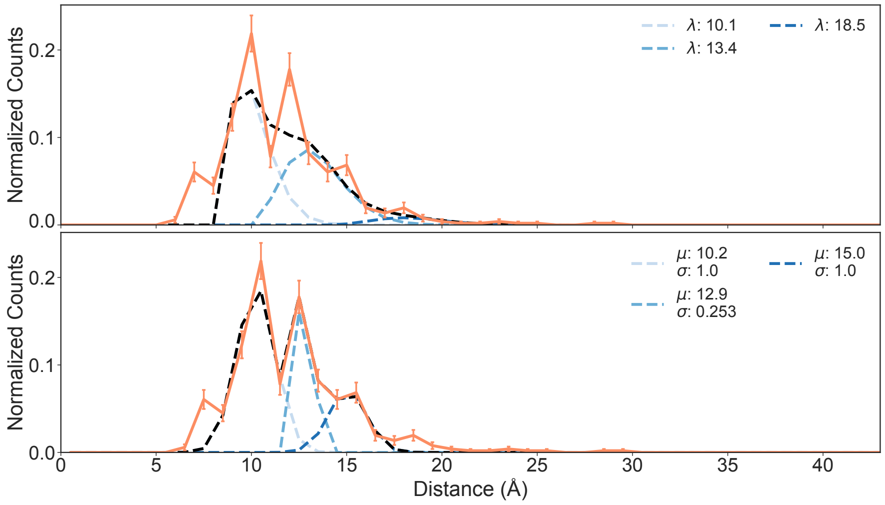

Figure S17: Fitting multiple peaks in the distribution of breaking distances. The dataset is the 10^{-6} Å/fs from the NEP model. The top plot fits multiple Gaussians according to Equation 9 and the bottom plot fits multiple Poisson distributions according to Equation 10.

These fits are very dependent on the input parameters which is why we refrain from fitting the rest of the data. Furthermore, we are mostly interested in the change in the average breaking distance which is why a single peak-fit (as done in the manuscript) is sufficient.

17 Push-pull of Au junctions

Similar to the push-pull experiments described in "Push-pull experiment with 4,4'-bipyridine", we perform push-pull simulations with pure Au junctions. We apply a constant speed of 10^{-7} Å/fs for both pushing and pulling. We check for rupture every 100,000 steps. When a rupture is detected, we stop pulling, run the simulation for an additional 5,000 steps, and then begin pushing. During pushing, we stop the simulation once the electrodes are brought back into contact.

In Figure S18, we show the distribution of the distance required to reform the junctions in Figure S18A and the correlation between the breaking and reformation distances in Figure S18B.

On average, the reformation distances in our simulations are slightly longer than those observed experimentally.[S17] This may be due to the much faster pulling rate in our simulations, which likely limits tip relaxation and melting compared to experiments.

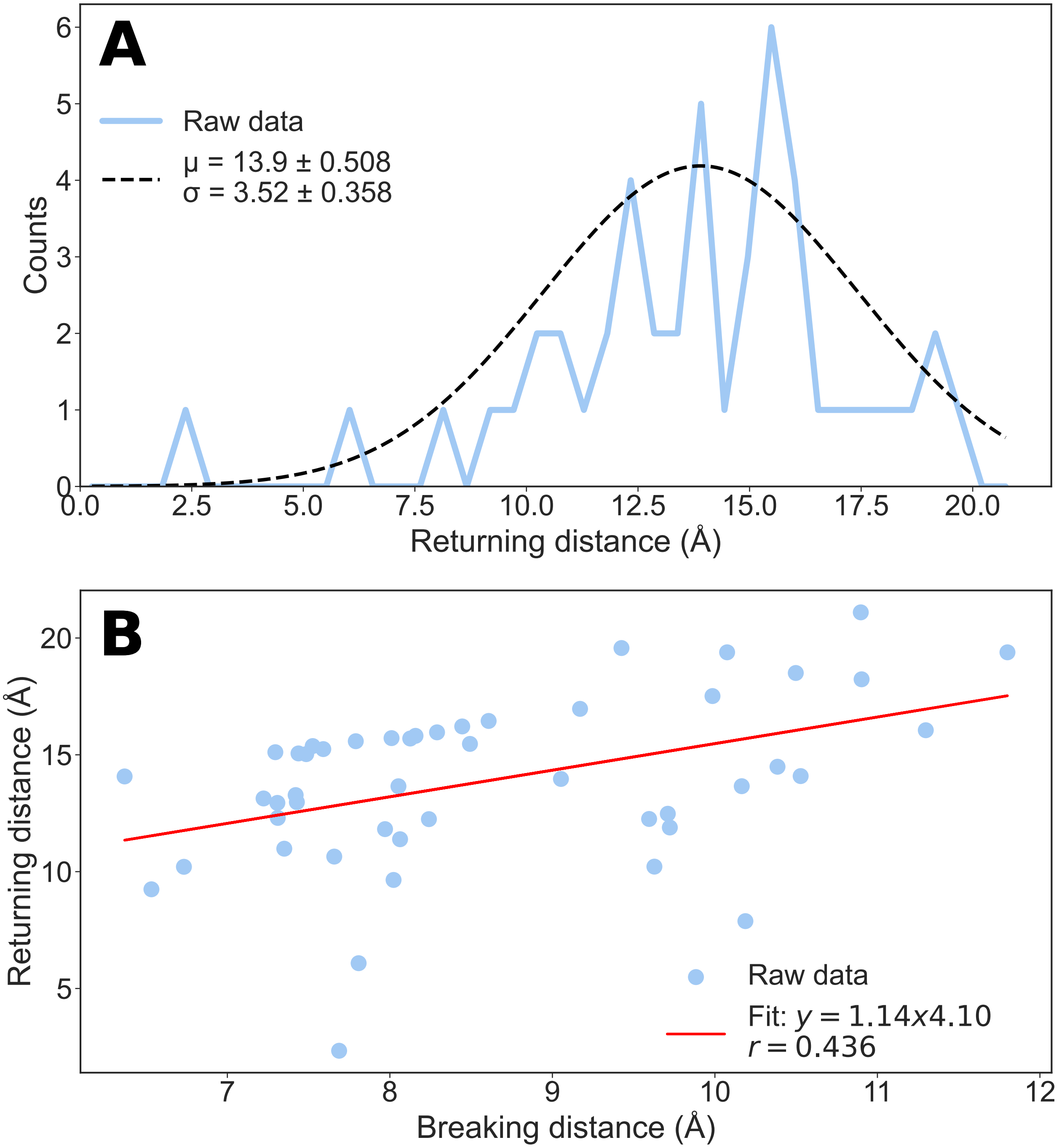

Figure S18: The distance to reform a junction. (A) Distribution of the distance from junction rupture to gold reform. The black dashed line represents a Gaussian fit; the estimated parameters are shown in the legend. (B) Distance required to break a junction plotted against the distance required to reform it. The red line indicates a linear fit; the estimated parameters are shown in the legend. The Pearson correlation coefficient, r, is also reported.

18 1D-histograms of forces

In Figure S19 and Figure S20, we show 1D-histograms of the forces from a single run with each of the investigated force fields: NEP model (green and orange lines), EAM potential (grey line), and ReaxFF (pink line). We have excluded the forces from all layers of the electrodes (600 atoms in total) to focus on the central region. We show the forces for the NEP model at two different pulling speeds. It is obvious that the forces from the ReaxFF potential differs substantively compared with the other two potentials. This might be because the parameterization set used for ReaxFF has not been trained to replicate the situation of amorphous Au. Instead, because the central region sits between the electrodes, the crystalline structure is imposed on the central region as well.

The distribution of forces from the NEP model and the EAM potential are very similar. Notably, for the NEP models, the X-components is, in general, slightly closer to 0 eV/Å. Conversely, the opposite seems to be true for the Z-components where they appear to be slightly closer to 0 eV/Å for the EAM potential.

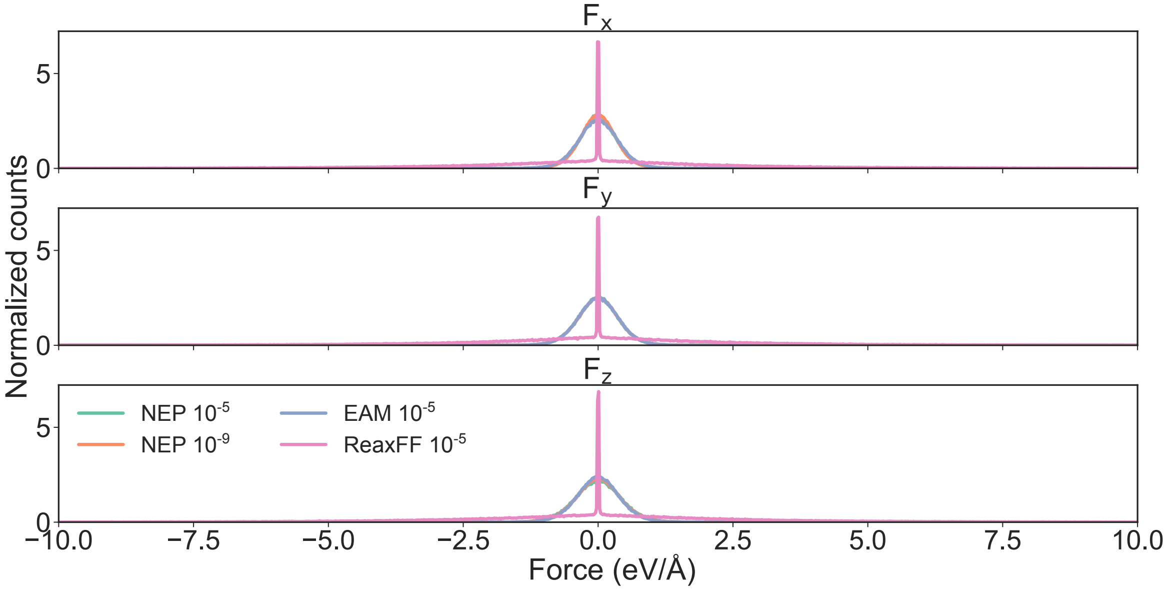

Figure S19: 1D-Histogram of forces in the X-, Y-, and Z-direction (top, middle, and bottom, respectively) for each the investigated force fields. (green) NEP at a pull speed of 10^{-5} Å/fs, (orange) NEP at a pull speed of 10^{-9} Å/fs, (grey) EAM at a pull speed of 10^{-5} Å/fs, and (pink) ReaxFF at a pull speed of 10^{-5} Å/fs. Each histogram has been normalized by the amount of samples in each trajectory.

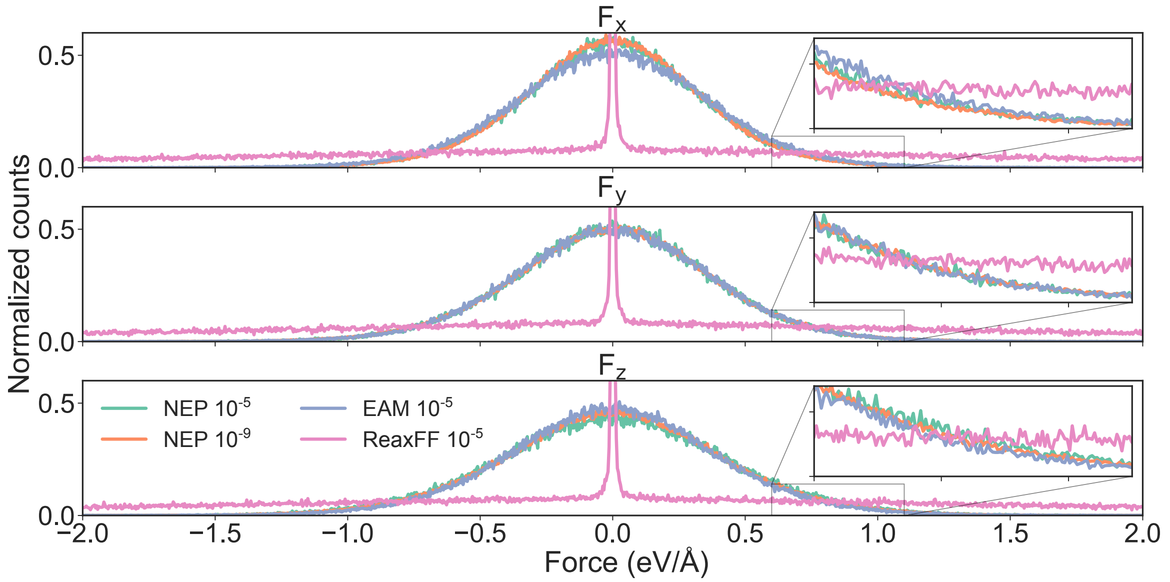

Figure S20: 1D-Histogram of forces in the X-, Y-, and Z-direction (top, middle, and bottom, respectively) for each the investigated force fields zoomed in compared with Figure S19. (green) NEP at a pull speed of 10^{-5} Å/fs, (orange) NEP at a pull speed of 10^{-9} Å/fs, (grey) EAM at a pull speed of 10^{-5} Å/fs, and (pink) ReaxFF at a pull speed of 10^{-5} Å/fs. Each histogram has been normalized by the amount of samples in each trajectory. Insets show the difference between the forces from the NEP model and the EAM potential.

References

(S1) Fan, Z.; Wang, Y.; Ying, P.; Song, K.; Wang, J.; Wang, Y.; Zeng, Z.; Xu, K.; Lindgren, E.; Rahm, J. M.; Gabourie, A. J.; Liu, J.; Dong, H.; Wu, J.; Chen, Y.; Zhong, Z.; Sun, J.; Erhart, P.; Su, Y.; Ala-Nissila, T. GPUMD: A package for constructing accurate machine-learned potentials and performing highly efficient atomistic simulations. *J. Chem. Phys.* **2022**, *157*, 114801.

(S2) Birch, F. Finite Elastic Strain of Cubic Crystals. *Phys. Rev.* **1947**, *71*, 809–824.

(S3) Davey, W. P. Precision Measurements of the Lattice Constants of Twelve Common Metals. *Phys. Rev.* **1925**, *25*, 753–761.

(S4) Tavazza, F.; Smith, D. T.; Levine, L. E.; Pratt, J. R.; Chaka, A. M. Electron Transport in Gold Nanowires: Stable 1-, 2- and 3-Dimensional Atomic Structures and Noninteger Conduction States. *Phys. Rev. Lett.* **2011**, *107*, 126802.

(S5) Tsutsui, M.; Morikawa, T.; Arima, A.; Taniguchi, M. Thermoelectricity in atom-sized junctions at room temperatures. *Sci. Rep.* **2013**, *3*, 3326.

(S6) Evangeli, C.; Matt, M.; Rincón-García, L.; Pauly, F.; Nielaba, P.; Rubio-Bollinger, G.; Cuevas, J. C.; Agraït, N. Quantum Thermopower of Metallic Atomic-Size Contacts at Room Temperature. *Nano Lett.* **2015**, *15*, 1006–1011.

(S7) Quek, S. Y.; Kamenetska, M.; Steigerwald, M. L.; Choi, H. J.; Louie, S. G.; Hybertsen, M. S.; Neaton, J. B.; Venkataraman, L. Mechanically Controlled Binary Conductance Switching of a Single-molecule Junction. *Nat. Nanotechnol.* **2009**, *4*, 230–234.

(S8) Magyarkuti, A.; Balogh, Z.; Mezei, G.; Halbritter, A. Structural Memory Effects in Gold–4,4-Bipyridine–Gold Single-Molecule Nanowires. *J. Phys Chem. Lett.* **2021**, *12*, 1759–1764.

(S9) Kamenetska, M.; Quek, S. Y.; Whalley, A. C.; Steigerwald, M. L.; Choi, H. J.; Louie, S. G.; Nuckolls, C.; Hybertsen, M. S.; Neaton, J. B.; Venkataraman, L. Conductance and Geometry of Pyridine-Linked Single-Molecule Junctions. *J. Am. Chem. Soc.* **2010**, *132*, 6817–6821.

(S10) Yanson, A. I.; Bollinger, G. R.; van den Brom, H. E.; Agraït, N.; van Ruitenbeek, J. M. Formation and manipulation of a metallic wire of single gold atoms. *Nature* **1998**, *395*, 783–785.

(S11) Untiedt, C.; Yanson, A. I.; Grande, R.; Rubio-Bollinger, G.; Agraït, N.; Vieira, S.; van Ruitenbeek, J. Calibration of the length of a chain of single gold atoms. *Phys. Rev. B* **2002**, *66*, 085418.

(S12) Smit, R. H. M.; Untiedt, C.; Rubio-Bollinger, G.; Segers, R. C.; van Ruitenbeek, J. M. Observation of a Parity Oscillation in the Conductance of Atomic Wires. *Phys. Rev. Lett.* **2003**, *91*, 076805.

(S13) Singh, A. K.; Chakrabarti, S.; Vilan, A.; Smogunov, A.; Tal, O. Electrically Controlled Bimetallic Junctions for Atomic-Scale Electronics. *Nano Lett.* **2023**, *23*, 7775–7781.

(S14) Todorov, T. N.; Sutton, A. P. Force and conductance jumps in atomic-scale metallic contacts. *Phys. Rev. B* **1996**, *54*, R14234–R14237.

(S15) Rubio-Bollinger, G.; Bahn, S. R.; Agraït, N.; Jacobsen, K. W.; Vieira, S. Mechanical Properties and Formation Mechanisms of a Wire of Single Gold Atoms. *Phys. Rev. Lett.* **2001**, *87*, 026101.

(S16) Pobelov, I. V.; Lauritzen, K. P.; Yoshida, K.; Jensen, A.; Mészáros, G.; Jacobsen, K. W.; Strange, M.; Wandlowski, T.; Solomon, G. C. Dynamic breaking of a single gold bond. *Nat. Commun.* **2017**, *8*, 15931.

(S17) Kamenetska, M.; Widawsky, J. R.; DellAngela, M.; Frei, M.; Venkataraman, L. Temperature dependent tunneling conductance of single molecule junctions. *J. Chem. Phys.* **2017**, *146*, 092311.
